# Recasting the Catalytic Legacy of Reverse Water–Gas Shift Reaction: Rational Design Strategies for Selective CO_2_ Valorization

**DOI:** 10.1021/accountsmr.5c00275

**Published:** 2026-07-03

**Authors:** Muhammad Asif Nawaz, José Antonio Odriozola, Tomas Ramirez Reina

**Affiliations:** † Department of Inorganic Chemistry and Materials Science Institute, University of Seville-CSIC, 41092 Seville, Spain; ‡ College of Environmental Science and Engineering, Beijing Forestry University, 35 Qinghua East Road, Haidian District, Beijing 100083, P.R. China

## Abstract

Rising atmospheric CO_2_ and climate pressures have intensified interest in carbon capture and utilization (CCU). The reverse water–gas shift (RWGS) reaction offers a direct route to convert CO_2_ into CO, a versatile syngas component for Fischer–Tropsch, methanol synthesis, and other downstream processes, thereby supporting a circular carbon economy. RWGS is inherently challenging: it is endothermic and favored only at high temperatures (>600 °C), which promotes catalyst sintering and high energy demand, while at lower temperatures the competing Sabatier reaction dominates, producing CH_4_ over Ni. Catalyst selection further complicates implementation: noble metals (Au, Pt, Rh) offer high CO selectivity but are costly, whereas base metals (Ni, Fe, Cu) are more abundant yet prone to methanation, sintering, or phase instability. Industrial deployment also demands resilience under water-rich environments, high space velocities, and thermal cycling associated with variable renewable H_2_ supply.

This Account presents our efforts to address these challenges through rational catalyst design guided by three central principles: electronic modulation via promoters to control adsorption and reaction pathways; active phase engineering to tune catalytic functionality and suppress undesired reactions, and structural and interfacial design to enhance stability and durability under realistic conditions. These principles are systematically explored using Ni- and Fe-based catalysts as model systems for scalable CO_2_ valorization. Alkali-promoted Ni catalysts, particularly Cs-modified Ni/CeO_2_, demonstrate how electronic tuning and metal–support interactions suppress methanation and enable CO-selective RWGS across a wide temperature range. Phase engineering further transforms Ni into phosphide intermetallic (Ni_2_P, Ni_12_P_5_), introducing active sites that favor CO formation while minimizing hydrogenation pathways. Complementary strategies in Fe-based systems, including MOF-derived Fe/carbon composites and Fe–Cu bimetallics, highlight the role of metal–support and metal–metal interactions in stabilizing active phases and enhancing CO yields. To address deactivation, nanoscale architectural control, such as core–shell and yolk–shell structures, has been employed to mitigate sintering and carbon deposition. Beyond single-function catalysts, dual-function and switchable materials integrate CO_2_ capture with catalytic conversion and enable dynamic operation across RWGS, methanation, and dry reforming, providing flexibility under variable process conditions. Mechanistic insights derived from operando spectroscopy reveal how surface defects, promoters, and support redox properties govern reaction pathways, including formate-mediated and redox mechanisms, thereby linking catalyst structure to function. Collectively, these studies demonstrate how integrating electronic, compositional, and structural design strategies can reconcile the competing demands of activity, selectivity, and stability in RWGS catalysis. By bridging mechanistic understanding with practical engineering considerations, this work advances RWGS from a fundamental reaction toward a scalable platform for CO_2_ valorization. These insights provide a framework for the design of next-generation catalytic materials and highlight the broader potential of rational materials engineering in enabling sustainable carbon utilization technologies. Looking ahead, operando-guided discovery, AI-assisted materials design, and integration with renewable energy infrastructures will be critical to realizing its full potential.

## Graphical abstract

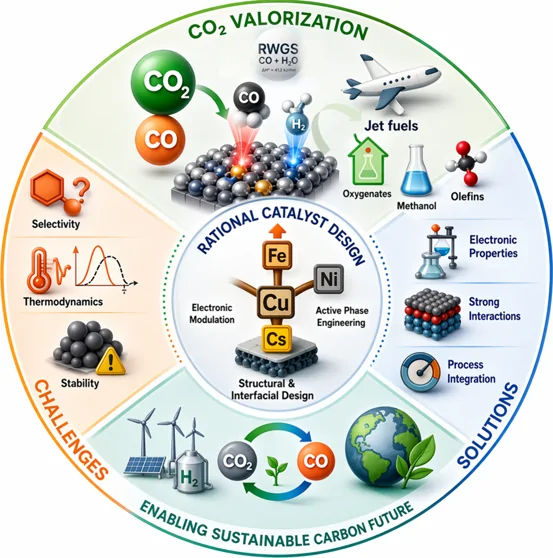

## 1 Introduction

The Reverse Water–Gas Shift (RWGS) reaction, giving a direct route to CO-rich syngas for downstream processes such as Fischer–Tropsch synthesis (FTS) and methanol synthesis (MTS), can be regarded as a vital part of sustainable CO_2_ valorization, especially when coupled with emerging green hydrogen infrastructures.^1–5^ However, despite this promise, the practical implementation of RWGS remains constrained by fundamental thermodynamic and kinetic challenges.^6–9^

As an endothermic reaction, RWGS is favored at elevated temperatures (>600 °C), where catalyst sintering, carbon deposition, and substantial energy demand become major limitations.^10,11^ At lower temperatures, the exothermic Sabatier reaction dominates, yielding CH_4_ rather than CO and reducing process utility. In addition, higher pressures can improve CO_2_ adsorption and activation at low temperature, but they also thermodynamically disfavor RWGS while promoting methanation, creating a persistent trade-off between conversion rate and CO selectivity. Figure S1 illustrates this complex dependence of CO_2_ conversion and CO/CH_4_ selectivity on pressure, temperature, and H_2_/CO_2_ ratio.^10,12,13^ Catalyst selection further underscores this tension, where noble metals (Au, Pt, Rh) offer high intrinsic activity and CO selectivity but are costly and scarce, while base metals (Ni, Fe, Cu) are economically attractive but prone to methanation, sintering, and phase instability.^14–21^ Industrial deployment adds further constraints, including operation under water-rich environments, high space velocities, and frequent thermal cycling associated with variable renewable hydrogen supply.^22,23^ Meanwhile, mechanistically, RWGS proceeds via multiple complex pathways (direct CO_2_ dissociation, H_2_-assisted, or redox) that depend on catalyst type, composition, and support environment.^24,25^ While CO_2_ activation as a rate-determining step, especially under low-temperature conditions, highlights the importance of catalysts that enhance adsorption and facilitate electron transfer to the stable CO_2_ molecule.^14,15,26^

In this context, our work targets efficient low-temperature Ni- and Fe-based RWGS catalysis by resolving activity-selectivity-stability trade-offs through metal–support interactions, defect engineering, active-phase optimization, and nanoscale control. These advances have relied on synergistic strategies involving alkali promoters (Cs, K, La) and engineered supports such as CeO_2_, TiO_2_, and MOF-derived matrices.^27–30^ Across these systems, the recurring design logic is consistent: electronic modulation, metal–support interfacial control, and nanoscale architecture are used to suppress methanation, minimize coking, and maintain long-term stability.^31–35^ Similarly, alkali-promoted Ni and Fe catalysts, nickel phosphides, and related multicomponent systems have demonstrated high CO_2_ conversion with low methane selectivity at moderate temperatures (250–300 °C).^36–39^ Dual-function materials (DFMs) and switchable catalysts further extend this concept by enabling dynamic operation across RWGS, methanation, and dry reforming pathways, thereby adapting catalyst function to variable hydrogen supply and downstream process requirements.^40,41^ This perspective collectively outlines our developments, which may position RWGS reaction as a pivotal step in the transition toward a resilient and circular carbon economy.

## 2 Engineering Excellence via Rational Design Strategies for Active Phase Selection of Ni–Fe-Based Catalyst Systems

The choice of active phase remains critical to CO_2_ valorization, where Fe and Ni have long been regarded as the “workhorses” owing to their affordability and intrinsic ability to activate both CO_2_ and H_2_. Acquiring higher activity requires balancing activity, selectivity, and stability. Where our approach has focused on “nobilizing” these base metals, seeking to emulate the selectivity of precious metals while maintaining the economic benefits of earth-abundant systems.

### 2.1 Electronic Modulation via Dopants: Cs as a Benchmark

Alkali promoters significantly influence Ni- and Fe-based catalysts through both structural and electronic effects. They stabilize key intermediates, enhance CO_2_ activation, and suppress undesired methanation. For example, Ni/CeO_2_ doped with K, Na, or Cs operates between 300 and 700 °C effectively, with methane formation suppressed at low temperatures while preserving intrinsic RWGS activity at higher ones.^15,42,43^ Spectroscopic studies attribute these improvements to increased Ni dispersion and the generation of oxygen vacancies in CeO_2_, thereby enhancing NiO reducibility and promoting CO_2_ activation. Cs-doped Ni/CeO_2_, in particular, delivers about 40% higher CO selectivity at 350 °C compared to its undoped counterpart. K offers a complementary benefit, where in low-temperature trials, careful adjustment of K:Ni ratios (0.5:10, 1:10, and 2:10) suppressed methanation and steered products toward CO, albeit with some loss of overall conversion.^44^ This compromise reflects the dual action of K, which both weakens hydrogenation pathways and stabilizes the Ni-CeO_2_ interface. Process simulations further confirm that such improvements can scale up to industrial syngas production. Among the alkalis, however, cesium consistently stands apart. Its strong electropositive character perturbs catalyst surfaces in ways that promote electron transfer from support or metal to adsorbed CO_2_, thereby easing activation. Across a wide spectrum of systems, Fe, Fe–Cu, Fe/Al_2_O_3_, β-Mo_2_C, Ni, and Pt-TiO_2_, Cs outperforms K and Na (Figure S2 and S3 **)** in both activity and selectivity (Cs > K > Na). Notably, it suppresses runaway methanation during cooling cycles, a feature highly valuable for downstream process integration. In Ni-based systems, Cs stabilizes active Ni sites, modulates interactions with CeO_2_, and promotes CO formation at low temperatures. The promoter effect was observed in both prereduced and as-prepared catalysts, indicating that alkali benefits are not solely dependent on pretreatment. Stability tests demonstrate that Cs-modified Ni catalysts maintain high CO selectivity and conversion across low- and high-temperature windows, even during extended operation. When combined with Cu in Fe–Cu catalysts, Cs enhances both activity and resistance to sintering via stabilizing metallic Fe^0^ and hindering carburization to Fe_3_C, achieving high CO_2_ conversion with near-complete CO selectivity at low temperatures. In Fe/Al_2_O_3_, moderate Cs loadings (2.5–5 wt %) optimize performance by improving reducibility, facilitating CO_2_ adsorption, and suppressing CH_4_ formation while maintaining long-term operational stability. Beyond Fe and Ni, Cs also exhibits remarkable versatility in β-Mo_2_C catalysts for enriching the surface electronically, promoting CO selectivity at low temperatures, and enabling in situ recarburization for stable operation. Similarly, on Pt-based catalysts, Cs alters the reaction mechanism, facilitating alternative low-temperature pathways such as formyl/acyl routes and frustrated Lewis pair (FLP)-assisted CO_2_ reduction, suppressing carbon deposition and achieving full CO selectivity at temperatures as low as 300 °C.^15^ It also acts as a structural promoter, enhancing nanoparticle dispersion and long-term stability. Its impact is both temperature- and composition-dependent, with low loadings favoring low-temperature CO_2_ conversion and higher loadings enhancing activity at medium-to-high temperatures. Overall, Cs functions as both an electronic and structural promoter: stabilizing active phases, strengthening CO_2_ adsorption and activation, suppressing side reactions, and enabling alternative mechanistic routes.

### 2.2 Multicomponent Systems: Bimetallic Synergies in Complex Formulations of RWGS Catalysts

The design of multicomponent catalyst systems expanding from alkali-promoted systems to the wider aspects of exploiting synergies between metals, promoters, and supports, offers a powerful route to enhancing CO_2_ conversion. Our various reports have consistently demonstrated that effective bimetallic and multicomponent blends often deliver performance that exceeds the sum of their individual parts, facilitating the application of synergistic design principles in RWGS systems.

#### 2.2.1 Ni-based Bimetallic Counterparts (Ni–Fe, Ni–Cr, Ni–Cu, Ni–Co, Ni–Ru, and Ni–P)

We have been complementary exploring the role of transition-metal oxide promoters on Ni catalysts supported on CeO_2_–Al_2_O_3_, a high-surface-area material with notable oxygen-buffering capacity. Among these systems, Fe and Cr exhibit contrasting effects: FeO_*x*_ significantly enhanced the catalyst’s activity, selectivity, and stability, while Cr_2_O_3_ exhibits different behaviors. Fe promotion markedly enhances activity, selectivity, and stability, whereas Cr tends to drive methanation. Ni–Fe/CeAl consistently achieved higher CO_2_ conversions than unpromoted Ni/CeAl and maintained performance even under high space velocities. This improvement stems from several factors: Fe promotes Ni dispersion, enriches Ni electronic density (as confirmed by XPS), and participates directly in RWGS via a redox cycle that generates additional CO-active sites. As a result, Ni-FeO_*x*_/CeAl combined high intrinsic activity with stability under demanding conditions, making it well-suited for compact reactor designs and dilute CO_2_ feeds. In contrast, Cr_2_O_3_ improved dispersion and texture but shifted the reaction pathway toward methanation, favoring formate-derived CH_4_ instead of CO. A similar story unfolds with alloying strategies. On saponite clay, Ni–Cu outperformed both Ni–Co and monometallic Ni.^45^ The Ni–Cu/saponite system sustained nearly complete CO selectivity over a wide temperature window, achieving ∼ 55% CO_2_ conversion with ∼ 80% CO selectivity. By comparison, Ni–Co and Ni catalysts both promote methane formation at lower temperatures, with Ni–Co showing intermediate behavior. The superior performance of Ni–Cu was attributed to alloy nanoparticles stabilized in a carbon-coated saponite matrix. During precursor decomposition, a thin in situ graphitic layer formed around the alloy, protecting it from coking and sintering. Alloying also altered the reaction network: while monometallic Ni tends toward the Sabatier route at low temperature, Ni–Cu retained CO selectivity even at 300 °C, effectively suppressing methanation.

Nickel phosphides represent a promising route to mitigate Ni’s tendency toward methanation, with activity and selectivity strongly dependent on both phase and support.^46^ In yolk–shell NiCo@SiO_2_, modest incorporation of Ni into Co spinel structures improved CO formation by creating modified Ni–Co sites. Likewise, Ni–Ru catalysts supported on ceria-zirconia dual-functional materials (DFMs) exhibited switchable behavior, operating across methanation, RWGS, and dry reforming pathways depending on reaction conditions, with stable cycling performance.^40,41^ These examples illustrate a broader principle: the effectiveness of bimetallic or multicomponent systems hinges on the mechanistic contribution of the second metal. Careful, mechanistically informed design is therefore essential for advancing Ni-based catalysts in CO_2_ valorization.

#### 2.2.2 Phase Control and Structural Tuning via Crystal Structure Sensitivity and Phase Evolution

We’ve systematically explored that crystalline phase and nanostructural control are as critical as the choice of active metal itself in the RWGS reaction. Where engineering the phase composition and creating unique architecture unlocks new avenues for optimizing activity, selectivity, and long-term stability.

Our studies show that crystalline phase and nanostructural control are as critical as the choice of active metal in the RWGS reaction. Engineering phase composition and designing tailored architectures offer powerful levers for optimizing activity, selectivity, and stability (Figure 1 and Figure S4).^24,36–39,47^ Such as in nickel phosphide systems, conventional metallic Ni strongly favors methanation, whereas specific phosphide phases (Ni_2_P, Ni_12_P_5_, Ni_3_P) display tunable selectivity governed by Ni/P ratio, support, and phase evolution across 300–800 °C. Ni-rich phases such as Ni_2_P and Ni_12_P_5_ deliver nearly complete CO selectivity over wide temperature ranges, while P-rich phases are less effective. Incorporation of phosphorus modifies Ni’s electronic structure, stabilizing CO intermediates and suppressing methane formation. On silica, Ni_2_P sustained >80% CO selectivity with excellent stability, while Ni_12_P_5_ supported on Al_2_O_3_ or Ce–Al exhibited reversible switching between RWGS and methanation through controlled phase transformations. DFT and microkinetic studies confirm that phosphidation (Ni_2_P, MoP) alters the reaction mechanism (COOH-mediated pathways vs direct CO_2_ dissociation). This shift lowers the activation barrier for CO formation while raising it for methane.^24,36^ Similar behavior is seen in Mo_2_C and MoP, which mimic precious-metal performance by producing almost exclusively CO.^39,47^ For instance, Cs-promoted Mo_2_C achieves 100% CO selectivity at low temperature, while MoP remains stable under high-conversion conditions. Even within nickel phosphides, subtle phase differences are decisive. Ni_2_P and Ni_12_P_5_ are highly selective, Ni_3_P allows partial methane formation, and NiP_2_ shows poor activity. Importantly, catalysts that undergo dynamic phase transitions, such as Ni_2_P partially converting to Ni_12_P_5_ during operation, retain high CO selectivity and stability, underlining the robustness of these materials.

**Figure 1:**
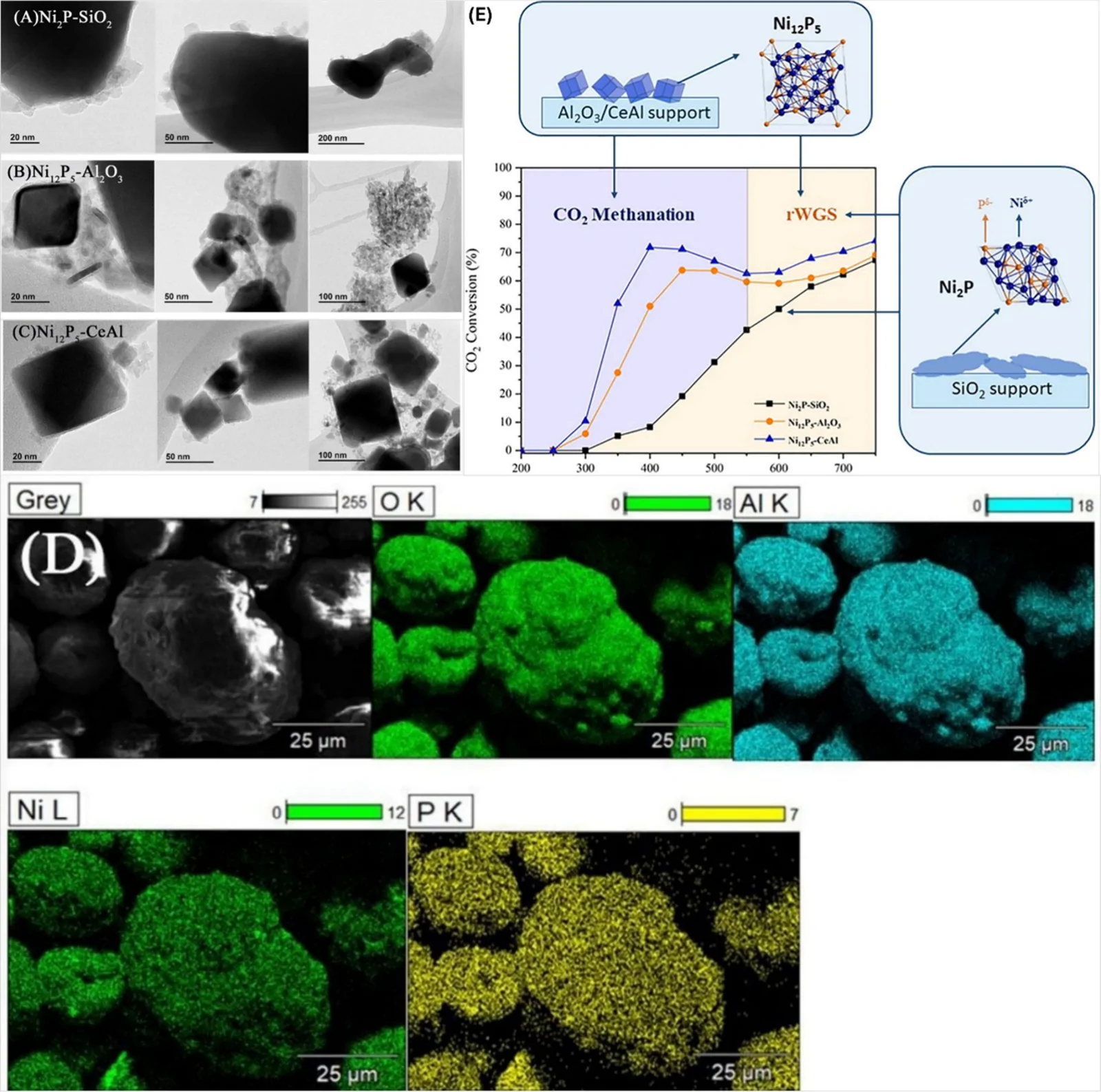
TEM/EDX micrographs, schematic illustration, and activity of different NiP catalysts. Reproduced with permission from ref 38. Copyright 2022 Elsevier. (a–c) TEM images of NiP catalysts. (d) SEM images with compositional mapping. (e) Schematic illustration and activity of different NiP catalysts.

Beyond crystalline phase engineering, we’ve found that nano structural control is a highly effective way to prevent the common problems of sintering and coking, which plague many catalysts. NiCo@SiO_2_ yolk–shell nanoreactors, with their confined bimetallic cores inside porous silica shells, led to exceptional CO selectivity. It was observed that even a small amount of nickel doping into the cobalt core can precisely tune the surface chemistry to favor CO over methane. The silica shell acts as a protective shield, not only stabilizing the nanoparticles against sintering but also minimizing carbon deposition. While perovskite-exsolved Ni–Rh socketed nanoparticles, Ni/*monoclinic*-ZrO_2_ active phase_,_ along with ZnO/Ni@m-SiO_2_ and Ni/ZnO@SiO_2_ yolk–shell architecture, though quadrupling active site populations and boosting CO_2_ conversion in DRM, can have dramatic performance in long-term performance RWGS operations.^31–33,48^ For instance, our NiCo@SiO_2_ catalysts showed no crystalline carbon after multiple reaction cycles, and a similar Ni@m-SiO_2_ system remained virtually coke-free after 140 h of operation at 850 °C. The confined environment of these structures may even enable new reaction pathways, with preliminary evidence suggesting the formation of C_2_^+^ products, which could be a powerful way to engineer selectivity toward higher-value chemicals. In conclusion, our research shows that by meticulously controlling a catalyst’s crystalline phase and nanostructure, we can create materials that are not only highly active and selective but also durable enough for long-term industrial applications. This approach allows us to go beyond simple material selection and design catalysts with a level of precision that was previously unattainable.

#### 2.2.3 Fe–Rh, Fe–Ni, and Fe–Cu Bimetallic Synergies

Fe-based catalysts have been core elements to our RWGS design strategies, owing to their redox flexibility and ability to accommodate diverse supports (CeO_2_, Al_2_O_3_, Ce–Al mixed oxides, MgAl_2_O_4_, and MOF-derived carbons).^49^ The performance of these systems, however, is strongly modulated by promoter incorporation and support interactions, which together determine phase stability, selectivity, and long-term resilience. Complementary to alloy-based strategies, MOF-derived Fe catalysts revealed that Rh dictated Fe particle growth (resulting nanoparticles) and favored highly dispersed Fe^0^-rich surfaces with the in situ route, showing the most promising behavior. Where, Rh doping enhancing the active Fe phase dispersion, boosts CO_2_ conversion at ≤ 500 °C, and suppresses CH_4_ formation.^50^ This work efficiently demonstrates the impact of Fe–Rh bimetallic counterparts in MOFs derived flexible precursors for both low- and high-temperature RWGS. As of Fe facilitating Ni in its main active component role, Ni also pays back well to Fe in Ni–Fe/MgAl_2_O_4_, conferring clear bifunctionality in RWGS and dry reforming of methane (DRM) systems.^51^ Structural analyses reveal that reduced catalysts form FeNi_3_ alloys whose size and dispersion are tuned by Ni loading. While Ni generally enhances CO productivity at elevated temperatures, particularly under CH_4_-rich feeds through the combined operation of RWGS and DRM, its role is double-edged. Excess Ni promotes coke deposition and, critically, at lower temperatures, shifts selectivity away from CO toward methanation. The 2 wt % Ni–Fe/MgAl_2_O_4_ composition strikes an optimal balance, producing finely dispersed FeNi_3_ particles, enhanced redox features, and sustained CO production with minimal carbon deposition. While focusing on the comparative impact on the stability in tentative approaches, Ni, though competing with others on elevated temperature, yet its methanation-prone nature at sub-500 °C, diverged the trends significantly to favor CO_2_ methanation, particularly at lower temperatures (Figure S5A **)**.

Since Fe–Cu systems offer a more favorable pathway for selective CO generation at intermediate and lower temperatures. The incorporation of Cu, as compared to the bare Fe/Al_2_O_3_ catalyst, markedly enhanced CO_2_ conversion and CO selectivity, attributing to the synergistic Fe–Cu interaction and improved resistance to sintering. While among the various formulations studied in a tentative approach, FeCu/CeO_2_–Al_2_O_3_ with a 0.25Fe-0.75Cu ratio emerged as particularly effective, delivering high CO_2_ conversion, near-complete CO selectivity, and excellent long-term stability (Figure S5B–S5D **)**. Where Fe–Cu/CeO_2_–Al_2_O_3_ catalysts exploit dynamic Fe–Cu oxide interactions, with Cu-doped ceria and Cu–Fe redox pairs (e.g., transient CuFe_2_O_4_), facilitating both H_2_ dissociation and CO_2_ activation. Unlike Ni, Cu does not strongly drive methanation; instead, it shifts selectivity toward nearly exclusive CO production. Further refinements through the Cu–Cs synergism in Cs–Fe/Al_2_O_3_ and Cs–Fe–Cu/Al_2_O_3_ systems resulted in the effective Fe–Cu platform with the basic nature of Cs, which facilitated electron transfer for enhanced CO_2_ adsorption. Interestingly, prereduced samples showed inferior performance compared to as-prepared catalysts, indicating that metallic Fe and Cu are not the dominant active phases. Instead, the metal/oxide redox pairs appear critical, offering the added process advantage of eliminating preactivation steps. In addition to its superior activity and selectivity, the combined effect of Cu or Cs promoters was instrumental in suppressing excessive carburization behavior in a tentative effort (discussed later) via phase control, inhibiting bulk (less active) Fe_3_C formation and stabilizing Fe^0^ while allowing limited Fe_5_C_2_ to appear under optimal conditions, a critical prerequisite for scale-up.^42^ Since combinations such as Fe–Cu, Ni–Fe, Ni–Ru, Ni–Cu, and Ni–P generate genuine synergies that enhance CO selectivity and stability, while others, such as Ni–Co or Ni–Cr, can divert the system toward methanation. Where both Ni and Cu are widely used as dopants in Fe-based systems, their roles are fundamentally different. Cu promotes redox (Mars–van Krevelen-type) pathways, enhancing oxygen mobility and stabilizing Fe oxide phases, thereby favoring CO selectivity. In contrast, Ni provides superior H_2_ activation, which is particularly beneficial under kinetically limited, low-temperature RWGS conditions, although it requires suppression of methanation pathways. Therefore, Ni and Cu should be considered complementary rather than competing promoters, where Cu enhances selectivity, and Ni improves reaction rates when appropriately engineered.

## 3 Hierarchical Manipulation of the Fe Nanostructure and Its Active Phase Probe

Iron-based catalysts highlight the importance of phase choice. Where, in Fe–Cu/CeO_2_–Al_2_O_3_ systems, operando studies revealed that CuFe_2_O_4_ spinel intermediates reduced in situ to metallic Fe and Cu, yet the partially reduced state proved more active than the fully metallic state. This suggests that redox pairs Cu^+^/Cu^0^ with Fe_3_O_4_/FeO or CeO_2_/Ce_2_O_3_, synergistically promote CO_2_ activation. Similar principles apply to Ni-FeO_*x*_/CeO_2_–Al_2_O_3_, where Ni exists as Ni^0^ while Fe remains as FeO_*x*_, creating an electron-rich Ni in close contact with oxygen-buffering ceria. While, despite decades of study, the identity of the true active phase in Fe-based RWGS remains debated. One of our key studies resolving this issue indicates that metallic Fe^0^ is more CO-selective than iron carbides, though a balance between Fe^0^ and Fe_5_C_2_ can enhance conversion (Figure 2).^42^ Fresh Fe catalysts, typically a mixture of Fe^0^ and Fe_3_O_4_, produced primarily CO, where over time, partial carburization led to Fe_5_C_2_, which increased CO_2_ conversion but introduced some CH_4_ selectivity as well. While Fe_3_C, by contrast, was the least desirable phase, both less active and less selective. Promoters such as Cu or Cs can suppress excessive carburization, inhibiting bulk Fe_3_C formation and stabilizing Fe^0^ while allowing limited Fe_5_C_2_ to appear under optimal conditions. The resulting steady-state mixture of Fe^0^ and Fe_5_C_2_ offers a productive compromise: high activity with predominantly CO selectivity. Interestingly, under ambient pressure, Fe_5_C_2_ also facilitated trace Fischer–Tropsch-type reactions, producing small hydrocarbons. Although not advantageous for RWGS, this observation opens the possibility of coupling RWGS and FT in a single multifunctional system if such reactivity could be directed. Since phase engineering, whether through stabilizing metallic vs oxide states, designing intermetallic phosphide/carbide phases, or nano structuring to resist sintering, emerges as a unifying principle for RWGS catalyst design.

**Figure 2:**
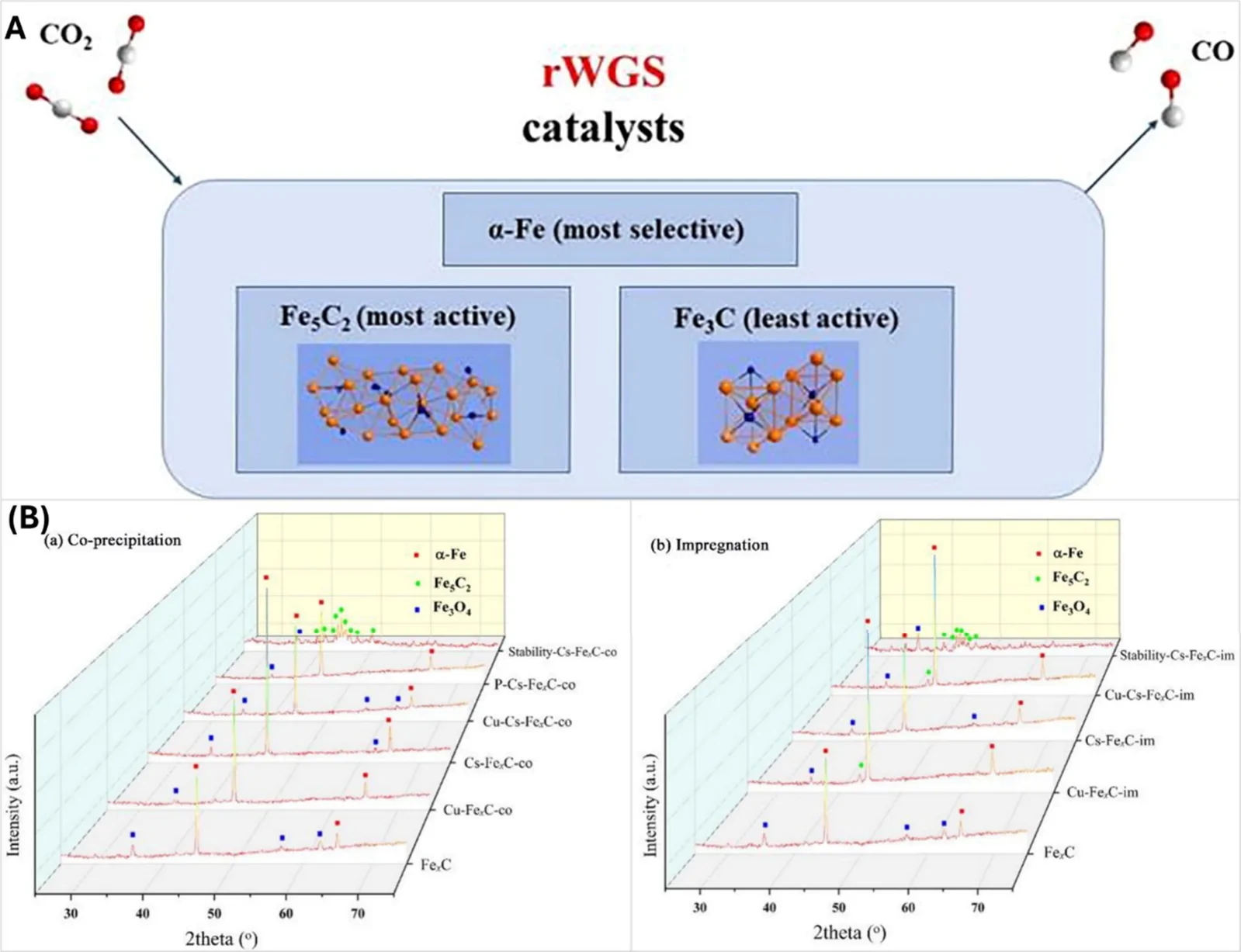
(A) Schematic illustration of different Fe phases and (B) XRD patterns of different spent samples prepared by coprecipitation and impregnation methods. Reproduced with permission from ref 42. Copyright 2022 Elsevier.

Meanwhile, on the legacy of designing structurally robust and highly dispersed carbon-supported Fe nanoparticles, MOF-derived Fe (C@FeRh*) nanoparticles embedded in a conductive matrix upon thermal treatment (1000 °C) (Figure 3A).^50^ The active phases obtained, α-Fe, Fe_3_C, and Fe_3_O_4_, were further tailored by Rh incorporation through two strategies: in situ doping and wet impregnation. The porous carbon matrix, typical of MOF-derived materials, further enhanced performance by stabilizing nanoparticles, providing high surface area, and promoting redox cycles. The Rh-doped Fe/C architecture achieved ∼ 37% CO_2_ conversion at 500 °C with nearly doubled CO selectivity (100%) compared to undoped Fe, strongly suppressing methanation, particularly at lower temperatures. These results demonstrate that MOF-derived Fe catalysts, especially with trace promoter incorporation, are highly promising for both low- and high-temperature RWGS. In a parallel effort, we explored natural carbonaceous materials as sustainable supports. Sporopollenin exine capsules (SpECs), derived from pollen, were infused with Fe and pyrolyzed to produce graphene-encapsulated Fe/Fe_3_C nanoparticles (Figure 3B-3 **C**).^52^ Structural and spectroscopic analyses (XRD, Raman, XPS) confirmed tunable porosity, surface area, and graphitization depending on Fe loading, while excessive loading (>20 wt %) led to morphology loss and Fe aggregation. Where the optimal fabricated design of SpEC-Fe10%, displayed high CO selectivity for RWGS at elevated temperatures (>600 °C), without requiring additional promoters. The carbon shell preserved Fe^0^ sites, prevented sintering, and eliminated the need for passivation. Although tested over shorter durations, demonstrates a low-cost, sustainable approach to producing graphitic carbon-encapsulated Fe catalysts, highlighting the potential of SpECs as renewable supports for CO_2_ valorization.

**Figure 3:**
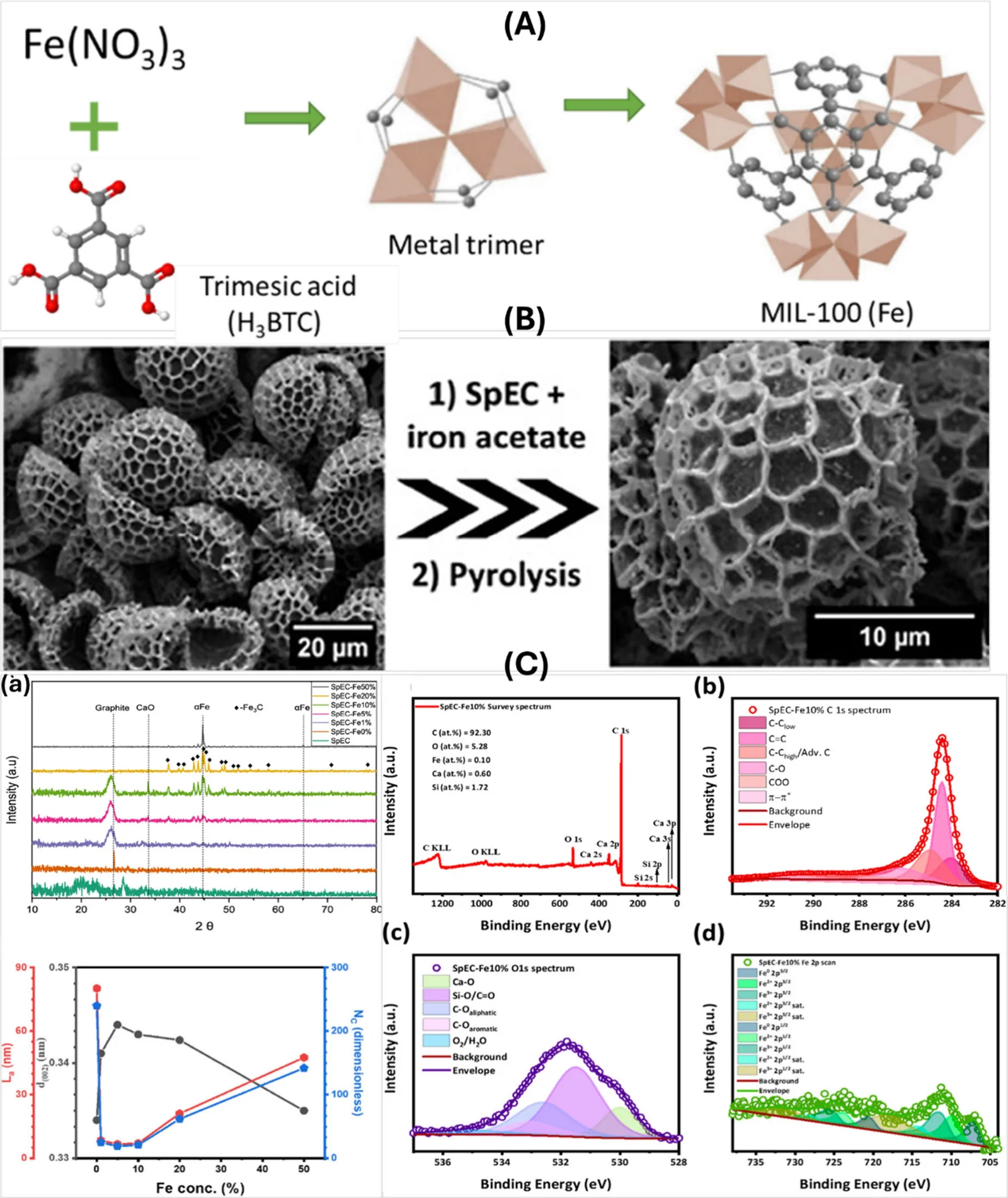
(A) Schematic synthesis of MIL-100 (Fe). Reproduced with permission from ref 50. Copyright 2023 Elsevier. (B, C) Schematic illustration, surface morphology, XRD, and XPS spectra of the SpEC-Fe catalyst. Reproduced with permission from ref 52. Copyright 2023 American Chemical Society.

## 4 Switchable and Dual-Function Materials (DFMs): Flexibility Across Multiple Reactions

Ni–Ru/CeO_2_–Al_2_O_3_ catalyst exhibits remarkable versatility by catalyzing three distinct CO_2_ conversion reactions, RWGS, CO_2_ methanation, and DRM, depending on the operating conditions. This “switchable” behavior allows the catalyst to selectively produce CO, CH_4_, or syngas, offering dynamic adaptability for diverse process requirements. The synergistic interaction between Ni and Ru underpins this multifunctionality. Nickel contributes its strength in CH_4_ activation and DRM, while ruthenium provides superior H_2_ activation and methanation performance (Figure 4 and Figure 5).^40,41^ The CeO_2_–Al_2_O_3_ support further enhances catalytic efficiency by combining the high surface area of alumina with the oxygen-buffering capacity of ceria, facilitating the oxidation of deposited carbon and maintaining particle dispersion. Small Ni nanoparticles stabilized on Ce–Al favor methanation, whereas Ru improves Ni reducibility and dispersion, enhancing overall activity. Under methanation conditions, the catalyst achieves near-equilibrium CO_2_ conversion to CH_4_ at relatively mild temperatures around 350 °C. In RWGS mode, it delivers high CO yields at moderate temperatures, demonstrating selective suppression of methane formation. At elevated temperatures (∼800 °C) in DRM mode, Ni dominates CH_4_ cracking while Ru facilitates carbon gasification, mitigating coke accumulation. Long-term DRM operation revealed gradual deactivation due to coking; however, the system can be regenerated through oxidative cycling, suggesting a cyclic operational strategy wherein the catalyst alternates between DRM and RWGS/methanation to maintain activity. While phase integration can also be exploited at the reactor scale. Here, Ni and Ru, acting as distinct active phases, while the Ce–Al support buffers oxygen and stabilizes dispersion, can extend the functionality to DFMs by combining CO_2_ sorbent phases (Na_2_O, K_2_O, CaO) with catalytic phases (Ni–Ru/CeAl).^53^

**Figure 4:**
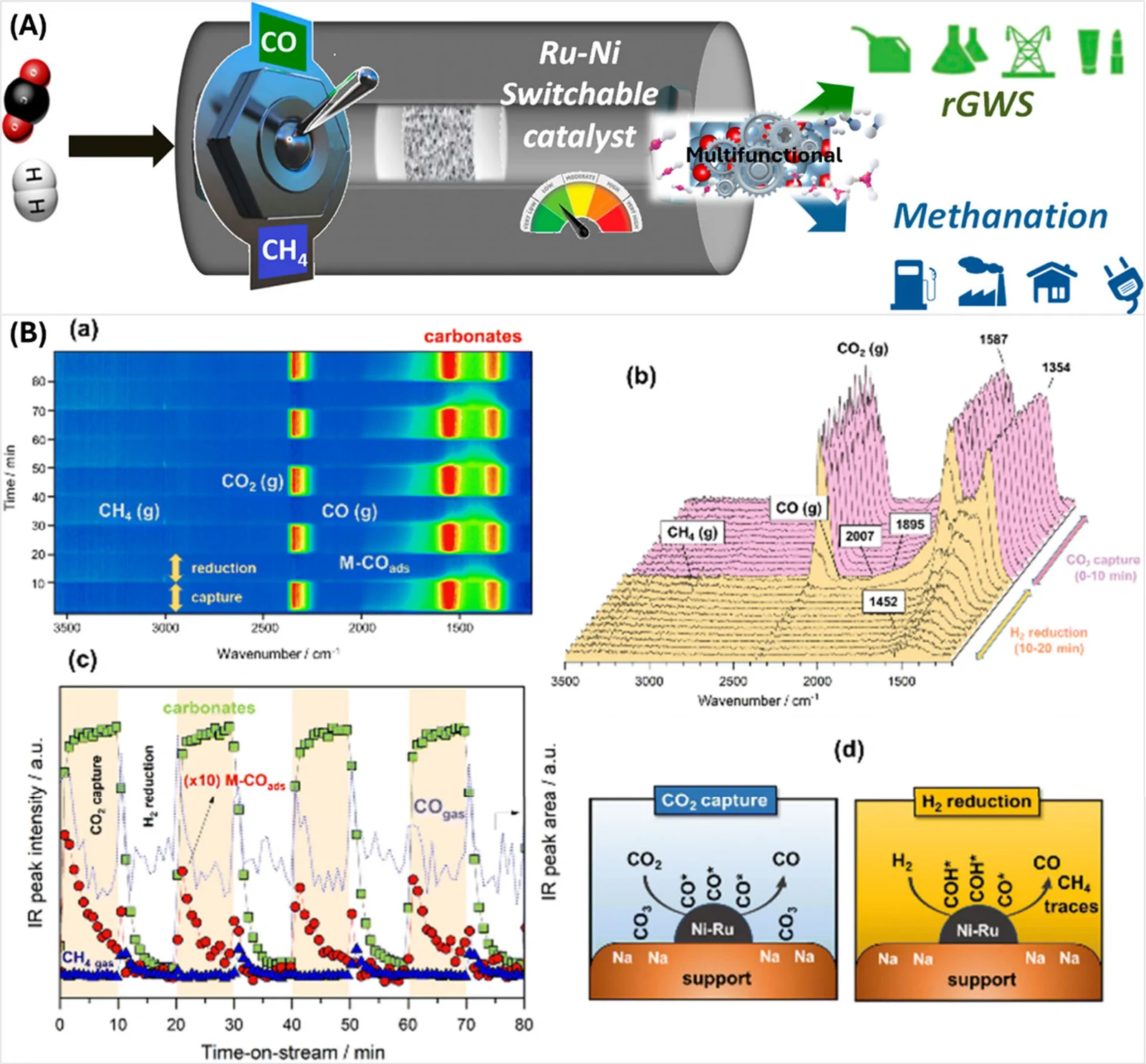
(A) Schematic illustration and (B) representative evolution of DRIFTS/IR spectra on the reduced NiRu/CeZr and NiRuNa/CeAl DFM during capture/reduction cycles. Reproduced with permission from ref 41 and ref 54. Copyright 2020 American Chemical Society and Copyright 2023 Royal Society of Chemistry.

**Figure 5:**
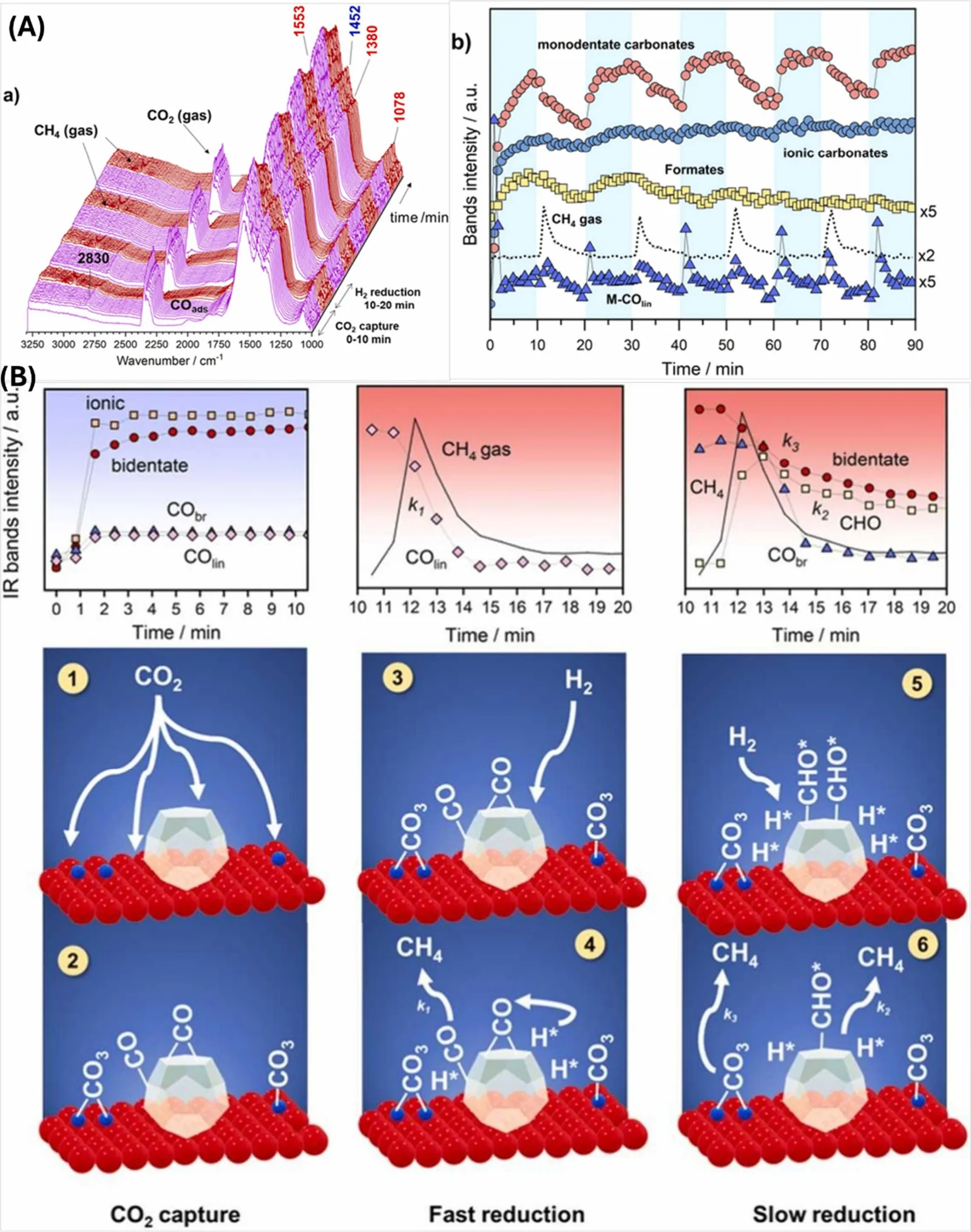
(A) Illustrative sketch of the reaction pathways involved in the capture/reduction cycles on NiRuCa DFM under model/realistic conditions and (B) the representative evolution of DRIFT/IR spectra during the successive cycle of capture/reduction. Reproduced with permission from ref 55. Copyright 2025 Elsevier.

To bridge CO_2_ capture and utilization, our group has developed Ni–Ru/alkaline-earth oxide/Ce–Al DFMs that seamlessly combine sorption and catalytic hydrogenation. These materials integrate CO_2_ sorbents such as Na_2_O, K_2_O, or CaO with active Ni–Ru sites, enabling CO_2_ to be captured as carbonates and subsequently converted under hydrogenating or reforming conditions directly into CH_4_, CO, or syngas. For instance, NiRuCa DFMs can sequester 338 μmol of CO per kg_DFM_ in DRM, 58 μmol of CO per kg_DFM_ in RWGS, and 104 μmol of CH_4_ per kg_DFM_ in CO_2_ methanation, depending on the reaction environment. Operando analyses revealed that carbonate species formed on the sorbent surface spill over to adjacent metal sites, where they undergo hydrogenation via formate-like intermediates.^54^ Fine-tuning the catalyst-sorbent interface proved critical, as it governs both capture efficiency and conversion kinetics.^55^ By integrating capture and reaction in a single material, this strategy eliminates intermediate CO_2_ release and compression steps, reducing the energy footprint. Moreover, such DFMs enable modular and distributed CCU applications, allowing CO_2_ to be adsorbed from dilute sources during off-peak hours and converted to fuels or syngas when renewable hydrogen is available. Since, this switchable functionality presents integrated multifunctional metal synergy with a tailored support design, where Ni–Ru/CeO_2_–Al_2_O_3_ exemplifies a new paradigm in adaptive catalysis for sustainable carbon utilization.

## 5 Mechanistic Insights from Operando Studies: Support-Promoter Effects Dictating Surface Chemistry and Reaction Pathways

By integrating operando spectroscopies (DRIFTS, UV–vis, NAP-XPS, XAS) with diffraction techniques and DFT modeling under conditions closely replicating the true reaction environment, we have been able to decisively probe whether CO_2_ conversion follows an associative pathway via formate intermediates or proceeds through dissociative redox steps.^56,57^ On Au/Al_2_O_3_ and various Ni-based catalysts, operando DRIFTS consistently detected bidentate formates, confirming their central role.^14,58^ The ultimate fate of these species, however, varied dramatically with catalyst composition. For instance, on Ni/ZrO_2_, formates were hydrogenated to methane, while on Ni–Cu alloys or Ni phosphides, the reaction stopped at CO, demonstrating a shift in selectivity. DFT results corroborated these findings, showing that Ni_2_P stabilized the COOH* intermediate enroute to CO, whereas MoP promoted direct CO_2_ dissociation.^24^ Carbonates also emerged as important intermediates on oxide supports such as CeO_2_ and MgAl, or within DFMs. The reducibility of the support proved to be equally decisive. In Au/TiO_2_, operando UV–vis revealed the dynamic generation of Ti^3+^ sites, oxygen vacancies that act as CO_2_ activation centers (Figure 6).^14^ DRIFTS captured hydroxycarbonyl-like intermediates, consistent with partial CO_2_ dissociation. A similar parallel mechanism operated on Pt/TiO_2_, where H_2_ spillover from Pt replenished vacancies and accelerated CO formation.^15^ In contrast, on inert Al_2_O_3_, Au followed a simpler formate pathway, yielding CO with negligible methane and accumulating formates on the surface. This highlights a duality in gold’s behavior, leading to selective CO formation via formates on inert oxides versus via vacancy-assisted redox chemistry on reducible supports. In both cases, high CO selectivity was maintained. MoO_3_ supported on the electronically rich Ti_3_AlC_2_ MAX phase illustrates the impact of support-mediated electronic effects (Figure 7).^59^ The MAX phase enhances MoO_3_ reducibility and oxygen vacancy density, enabling efficient electron transfer to CO_2_ antibonding orbitals, producing the highest intrinsic activity and complete CO selectivity.

**Figure 6:**
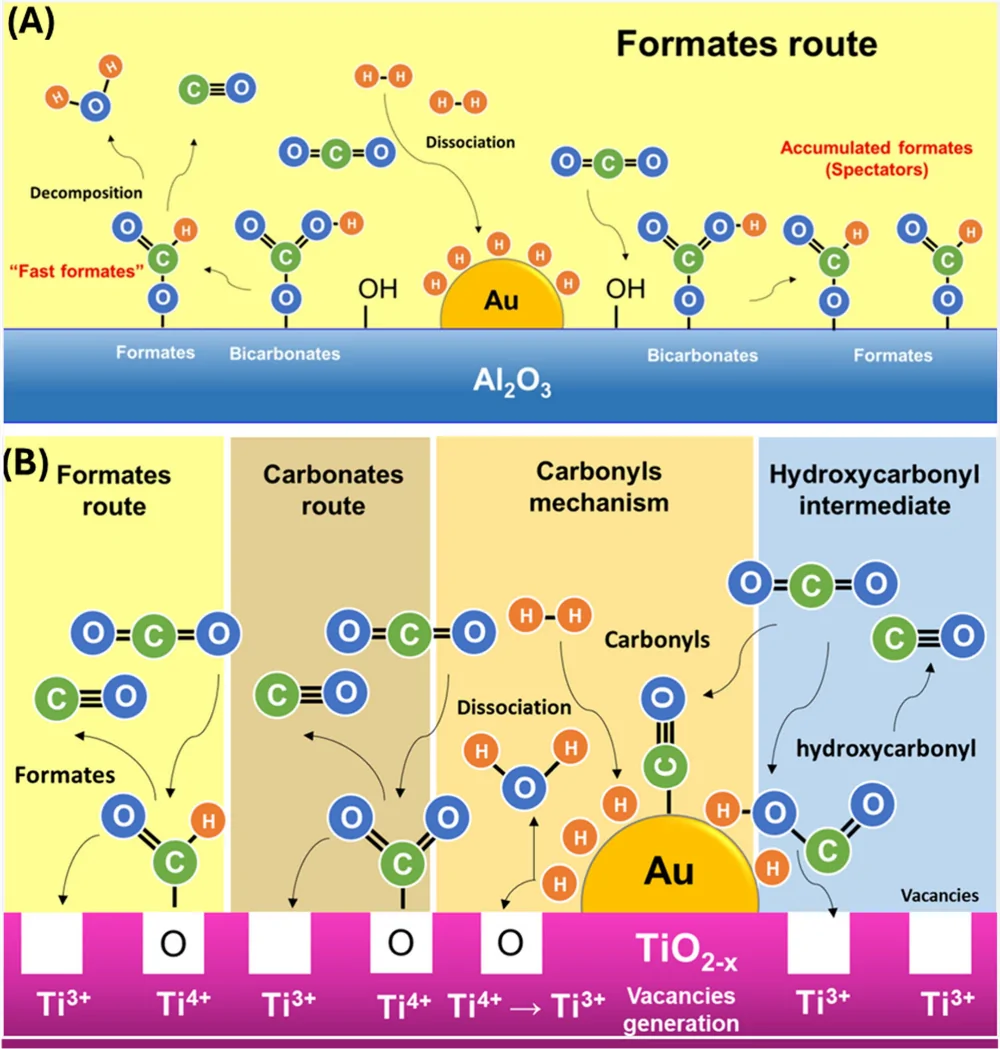
(A, B) Proposed RWGS reaction mechanism for the Au/Al_2_O_3_ and Au/TiO_2_ catalysts. Reproduced with permission from ref 14. Copyright 2018 American Chemical Society.

**Figure 7:**
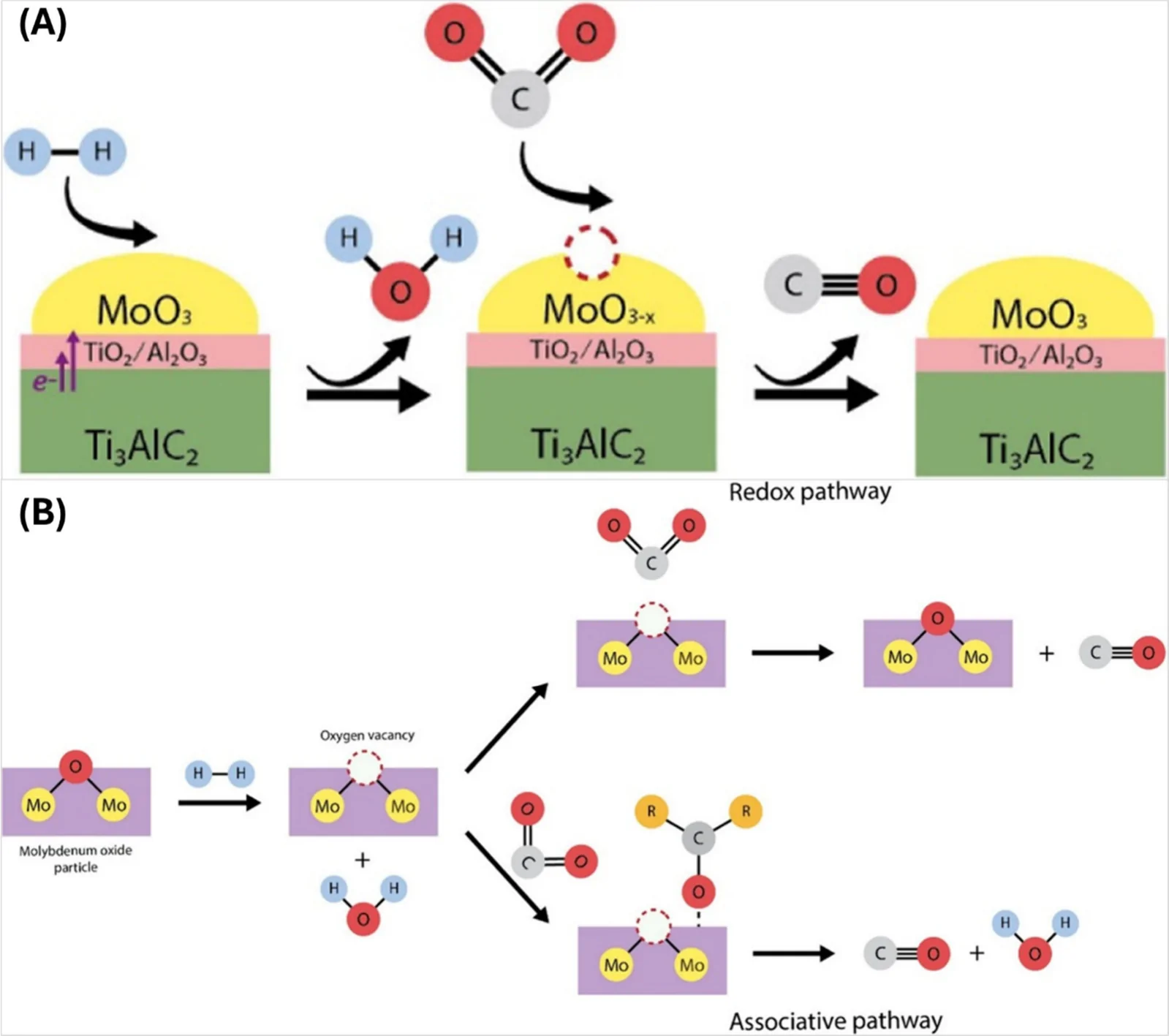
(A) Impact of oxygen vacancies on MoO_3_ particles and (B) the proposed RWGS reaction pathways. Reproduced with permission from ref 59. Copyright 2021 American Chemical Society.

Platinum, by contrast, often veered toward methanation under H_2_-rich feeds, a challenge we addressed by engineering subnanometric Pt/TiO_2_ catalysts promoted with cesium. Operando spectroscopy showed that cesium altered the reaction landscape by neutralizing surface hydroxyls, stabilizing ultrasmall Pt clusters, and enabling heterolytic H_2_ splitting through frustrated Lewis pair (FLP)-like sites (Figure 8).^15^ This promoted selective CO formation via formyl and acyl intermediates, suppressing methanation entirely at 300 °C. On Ni–Cu alloys, electron-rich interfaces weakened CO binding and prevented its further hydrogenation, while on unmodified Ni, stronger CO adsorption led to methane. Likewise, Pt/TiO_2_ alone produced methane, yet Cs-modified Pt/TiO_2_ channeled the reaction cleanly to CO. The FLP-type pathway was particularly instructive: a hydride generated from heterolytic splitting reacted directly with CO_2_ to form CO or formate, while a proton stabilized oxygen species, closing the cycle without producing surface carbon prone to overhydrogenation. Since it can be illustrated that reducible oxides like TiO_2_ and CeO_2_, for instance, contribute oxygen vacancies that are crucial for activating CO_2_. Similarly, alkali promoters such as cesium can increase the concentration of these vacancies and alter the surface’s basicity, all while fine-tuning the electronic structure of the metal itself. For example, we found that promoting Ni/CeO_2_–Al_2_O_3_ with FeO_*x*_ significantly boosted activity and CO selectivity compared to Ni/Al_2_O_3_ alone. The density of these oxygen vacancies is a delicate balance. Too few and the CO_2_ activation is hindered; too many and the catalyst risks over-reduction, leading to undesirable methane formation. Basic supports like MgO or alkali-promoted clays stabilize key carbonate intermediates, which channel the reaction toward CO. In contrast, acidic surfaces like Al_2_O_3_ can lead to overhydrogenation. In summary, our work shows that RWGS selectivity is not determined by the metal alone. It is the result of a precise interplay between the electronic structure, support reducibility, promoter chemistry, and the dynamics of surface vacancies. However, the upcoming studies would further enlighten the path forward for surface restructuring under working conditions, helping distinguish associative formate-type routes from redox pathways in CO_2_ hydrogenation and RWGS.

**Figure 8:**
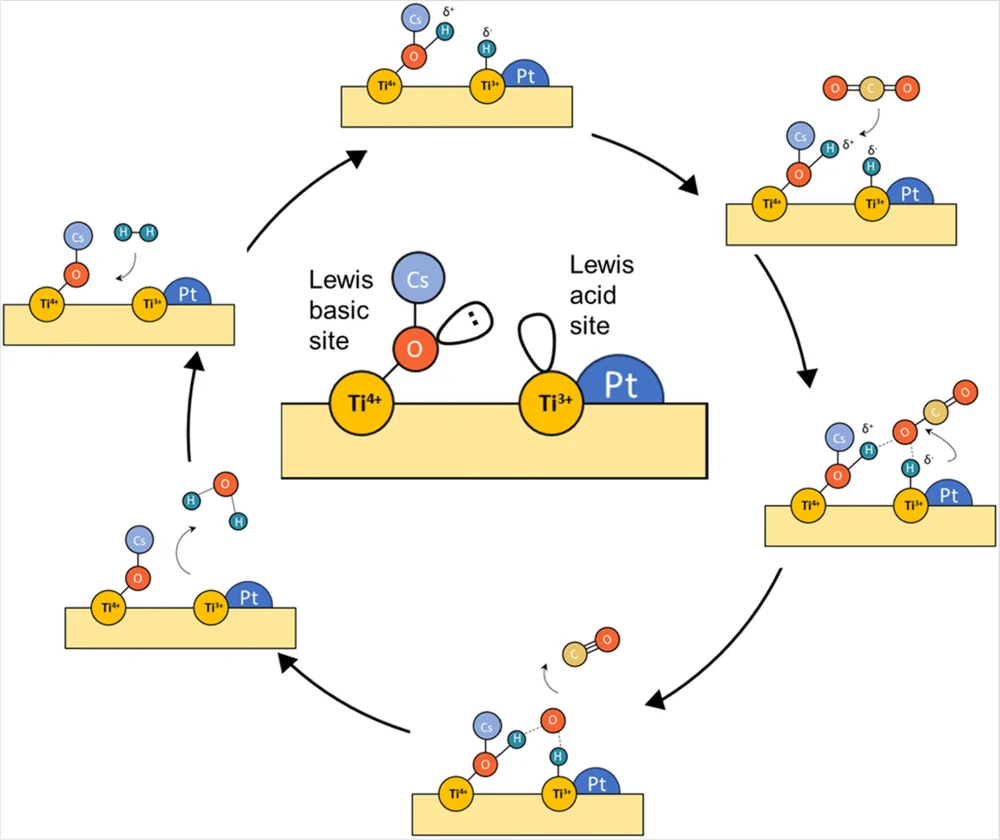
Proposed FLPs pathways for Pt–Cs/TiO_2_. Reproduced with permission from ref 15. Copyright 2024 Royal Society of Chemistry.

## 6 Combating Stability and Scalability in RWGS Catalysis with Integrated CO_2_ Utilization

Many of the most promising CO_2_-upgrading materials distinguish themselves not by a single attribute but through paired or layered functionalities. They combine long-term stability with operational flexibility or merge CO_2_ capture and catalytic conversion within a single, compact system. Such multitrait designs minimize traditional trade-offs between durability, selectivity, and process integration, enabling practical, low-footprint deployment. Importantly, these catalysts are engineered to dovetail with downstream processes, including Fischer–Tropsch synthesis, methanol production, and solid oxide fuel cells, facilitating energy-efficient, circular-carbon pathways. Nickel phosphides offer an alternative strategy, such as Ni_2_P/SiO_2_ achieves near-complete CO selectivity over broad temperatures while resisting coking and phase degradation, making it ideal for low-temperature RWGS streams (Figure S6A **)**. Ni_12_P_5_ phases on alumina or ceria-alumina provide switchable chemistry, catalyzing methanation at low temperatures but shifting to RWGS at higher ones, enabling modular, distributed power-to-X deployment. Architectural control presents yet another pathway. Yolk–shell and core–shell structures, such as Ni–Co@SiO_2_ (Figure S6B **)** and Ni-ZnO@m-SiO_2_, integrate mechanical robustness with catalytic tuning. Encapsulation limits sintering and filamentous carbon formation, while bimetallic interactions guide product distribution: Ni–Co favors CO, whereas Ni-ZnO excels in DRM for syngas. Confinement preserves active surface area and subtly modifies adsorption energetics, producing selective, stable catalysts whose syngas outputs are directly compatible with FT, methanol, or SOFC units. Fe-based catalysts add a further dimension via redox flexibility and phase control. Fe–Cu on CeO_2_–Al_2_O_3_ leverages dynamic Cu–O–Ce and Cu–Fe redox pairs to sustain high CO selectivity at intermediate temperatures while resisting overcarburization; Cs promotion enhances electron transfer and suppresses methanation. MOF-derived Fe/C catalysts from MIL-100­(Fe), especially with trace Rh doping, stabilize nanoparticles in a conductive carbon matrix, improving low-temperature CO_2_ conversion and minimizing CH_4_ formation. These designs naturally complement integration into downstream syngas conversion processes.

Some concepts unify capture, regeneration, and conversion within a single material. Ni–Ru DFMs with Ca/Na/K adsorbents reversibly store CO_2_ between 350 and 650 °C and release it as CH_4_, CO, or syngas on demand (Figure S6C **)**. Ni–Ru bimetallics exemplify this dual capacity. On Ce–Al supports, trace Ru improves Ni reducibility and dispersion, generating catalysts that are both durable and switchable: they produce CO via RWGS, CH_4_ via Sabatier chemistry, or syngas under dry reforming, depending solely on feed composition and temperature. When paired with CaO or alkali adsorbents, Ni–Ru evolves into DFMs capable of capturing CO_2_ and immediately converting it to CO or CH_4_. These integrated adsorbent-catalyst systems, validated with real fermentation gases, achieve regenerability, impurity tolerance, and cyclic operation in a single formulation (Figure S6D). Operando DRIFTS-MS studies reveal carbonate and carbonyl intermediates followed by hydrogenation, validating the integrated mechanism. These systems exemplify process intensification: one material, one cycle, and an output directly compatible with downstream chemical or energy platforms. Taken together, these examples illustrate a fundamental design principle: engineering catalysts to carry complementary functions, stabilizing active sites while enabling dynamic redox or adsorption behavior, rather than adding single-purpose traits piecemeal. Materials that marry durability with redox flexibility, capture with conversion, or confinement with selectivity directly address the stability, scalability, and integration challenges that have historically limited RWGS and broader CCU deployment (Figure 9).^55,60^

**Figure 9:**
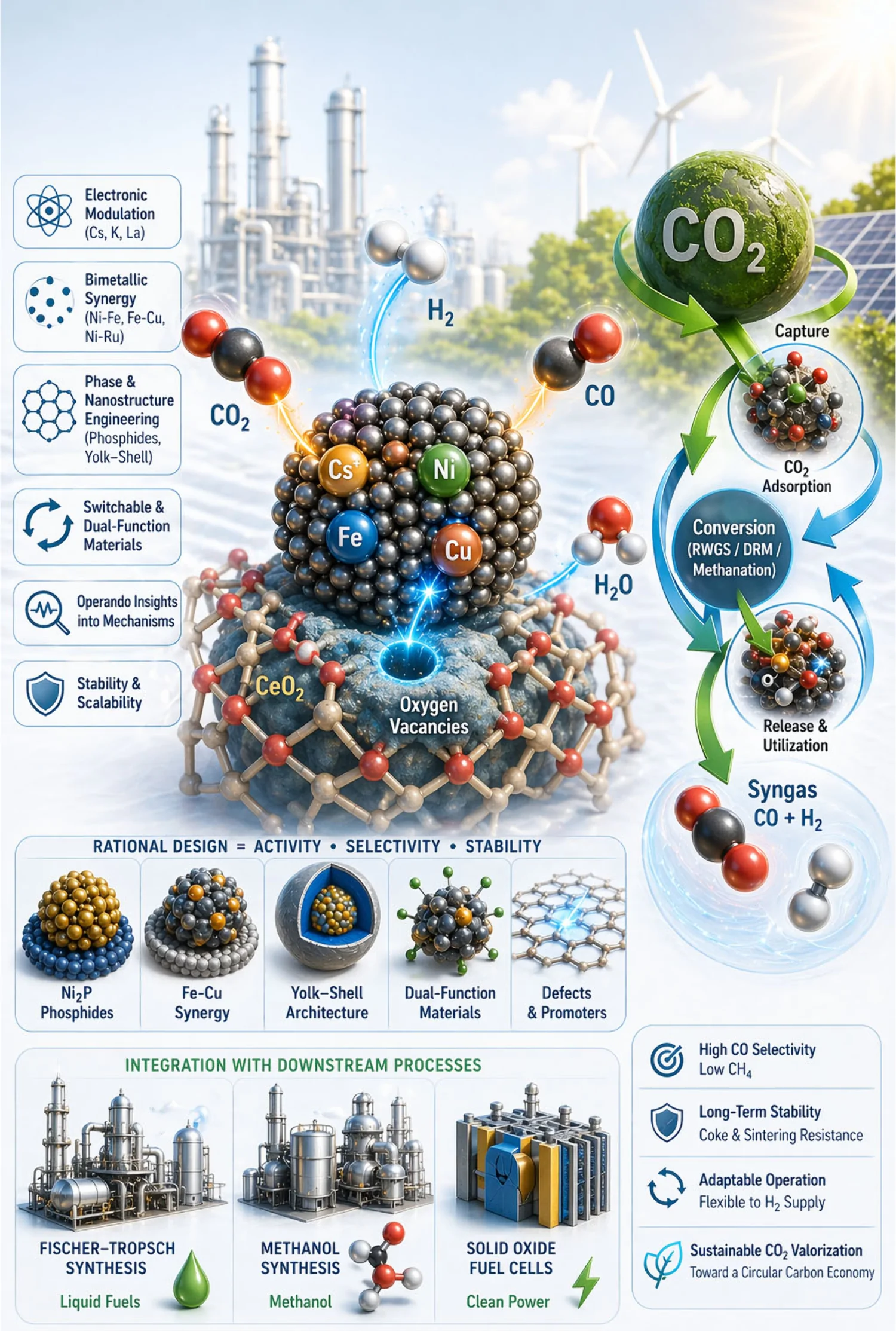
Schematic design strategies for selective, stable, and scalable RWGS catalysts.

## 7 Conclusions and Perspectives

This Account has highlighted rationally designed catalysts spanning Ni-, Cu-, and Fe-based systems, noble-metal promoters, nanostructured architectures, and multifunctional hybrids for CO_2_ valorization. By suppressing methane formation, stabilizing active phases, and enabling switchable or integrated functionality, these advances address long-standing barriers of selectivity, stability, and scalability. Alkali-promoted Ni/CeO_2_ catalysts, Ni phosphides, Fe–Cu–Ce systems, subnanometric Pt clusters on Cs-TiO_2_, and yolk–shell nanoreactors exemplify strategies that achieve the trifecta of high activity, CO selectivity, and resilience under relevant operating conditions. The design principles established with noble elements extend to base metals: Pt–Cs/TiO_2_ remains the most active and selective system, while Cu–Cs/TiO_2_ offers a cost-effective alternative with performance nearly comparable. Ni–Cs/TiO_2_ also benefits from Cs promotion, shifting product distribution toward CO, although its low-temperature intrinsic activity remains lower than that of Pt and Cu. Collectively, these results show that alkali promotion and reducible supports can “nobilize” base metals, narrowing the performance gap with noble-metal catalysts while preserving economic viability. Their activity correlates with metallic nanoparticles in proximity to oxygen vacancies, underscoring the importance of maintaining metal–support interfaces without excessive encapsulation.

Looking ahead, the most promising advances in RWGS catalysis will likely come from more sophisticated catalyst architectures, stronger deactivation resistance, and closer integration with renewable H_2_ supply chains. Operando spectroscopy and microscopy should continue to guide catalyst design by resolving active sites, dynamic restructuring, and transient intermediates, while combined time-resolved experiments and computational approaches will help establish mechanistic fingerprints for rapid materials screening. At the materials level, next-generation catalysts will benefit from hierarchical and confinement-based architecture, exsolved structures, and defect-rich supports that stabilize active phases under high-temperature, water-rich, and cycling conditions. Equally important is the need to translate these nanoscale advantages into scalable reactor formats such as pellets, monoliths, and structured beds without sacrificing performance. From a process perspective, RWGS will increasingly be designed as part of integrated CO_2_ utilization platforms, coupled to downstream Fischer–Tropsch, methanol, or dual-function conversion systems, and powered by intermittent renewable hydrogen. In this context, switchable catalysts and DFMs provide a particularly compelling route toward compact, flexible, and intensified CO_2_ conversion units. Finally, the exploration of new material classes, carbides, nitrides, borides, and perovskite-derived exsolved systems promises further breakthroughs. Just as Ni phosphides redefine selectivity and stability, other underexplored families may yield active phases with unique functionalities.

## Supplementary Material



## Data Availability

Data will be made available on request.

## Notes

Supplementary texts and figures for additional catalytic and characterization data (PDF)
