# Supplementary material for: Recasting the Catalytic Legacy of Reverse Water–Gas Shift Reaction: Rational Design Strategies for Selective CO2 Valorization
Source: Acc Mater Res. 2026 Jul 3;7(10):1018–33. doi: 10.1021/accountsmr.5c00275 (PMC13711629; doi:10.1021/accountsmr.5c00275)
Supplement: Supplementary file 1 [file mr5c00275_si_001.pdf]

Supporting Information

Recasting the Catalytic Legacy of Reverse Water-Gas Shift Reaction: Rational Design Strategies for Selective CO_2 Valorization

Muhammad Asif Nawaz [a,*], J.A Odriozola [a,b], and Tomás Ramírez Reina [a,*]

[a] Department of Inorganic Chemistry and Materials Science Institute, University of Seville-CSIC, 41092 Seville, Spain

[b] College of Environmental Science and Engineering, Beijing Forestry University, 35 Qinghua East Road, Haidian District, Beijing 100083, P.R. China

Correspondence: mnawaz@us.es | tramirez@us.es

Supplementary texts and figures for additional catalytic and characterization data.

Figure S1. Impact of reaction variables on CO_2 conversion and CO/CH_4 selectivity

Figure S2: Catalytic activity and stability run for different Ni/CeO_2, $Fe\text{-}Cu/Al_2O_3$, and Fe/Al_2O_3-based catalysts.

Figure S3:

A-D) Specific activity towards CO and CH_4 for different Pt and Cs-doped catalysts.

E-F) CO_2 conversion and stability tests of NiFe/CeAl and NiCr/CeAl catalysts at different conditions.

Figure S4:

A-C) TEM micrographs, SEM micrographs with EDX results, and catalytic activity of Mo-P-SiO_2.

D-E) Catalytic activity and schematic illustration of Cu and Cs promoted Mo_2C catalysts for RWGS. F) Comparative DFT illustration of MoP (0001) & Ni_2P (0001) surfaces for selected RWGS intermediates.

Figure S5:

A) Stability test for Fe/CeAl, FeNi/CeAl, and FeCu/CeAl Catalysts.

B) CO and CH_4 selectivity for Fe/Cu catalysts with different metal ratios.

C-D) CO_2 conversion of 0.25Fe0.75Cu after different pre-H_2-activation temperatures.

Figure S6:

A) Post-stability and post-reaction XRD patterns for $Ni_{12}P_5$-SiO_2 and Ni_2P-SiO_2 catalysts.

B) Schematic illustration & cyclability study of the NiCo@SiO_2 catalyst at 15,000 ml/g_{cat}h, H_2:CO_2 = 4. C) Feasibility study results of stable-switchable NiRuNa, NiRuK and NiRuCa DFMs. D) Stability run for NiRu/CeAl in different CO_2 conversion platforms (Methanation, RWGS, and DRM).

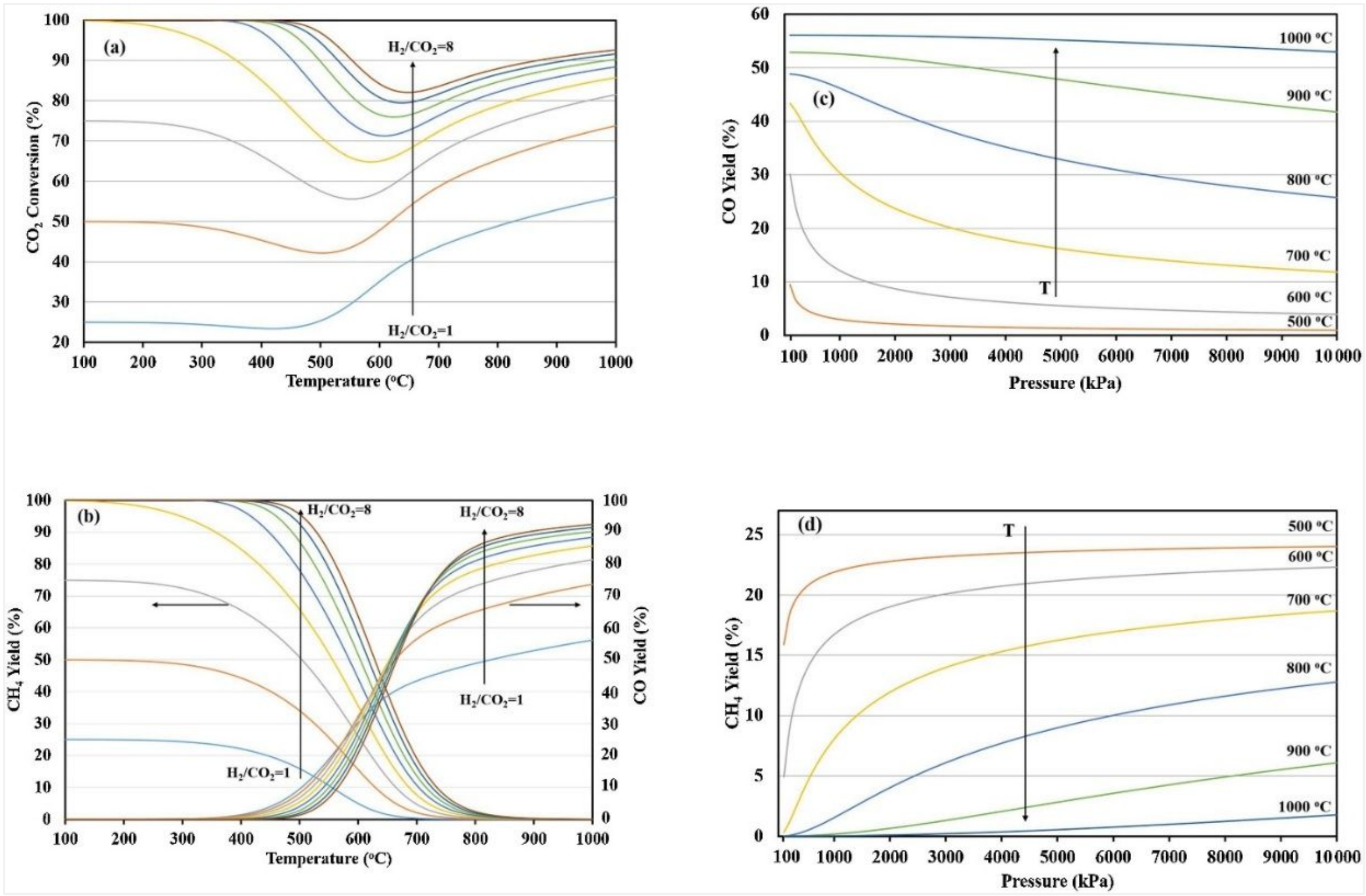

Figure S1. Impact of reaction variables on CO_2 conversion and CO/CH_4 selectivity, Reproduced with permission from ref. [1], Copyright 2019 Elsevier.

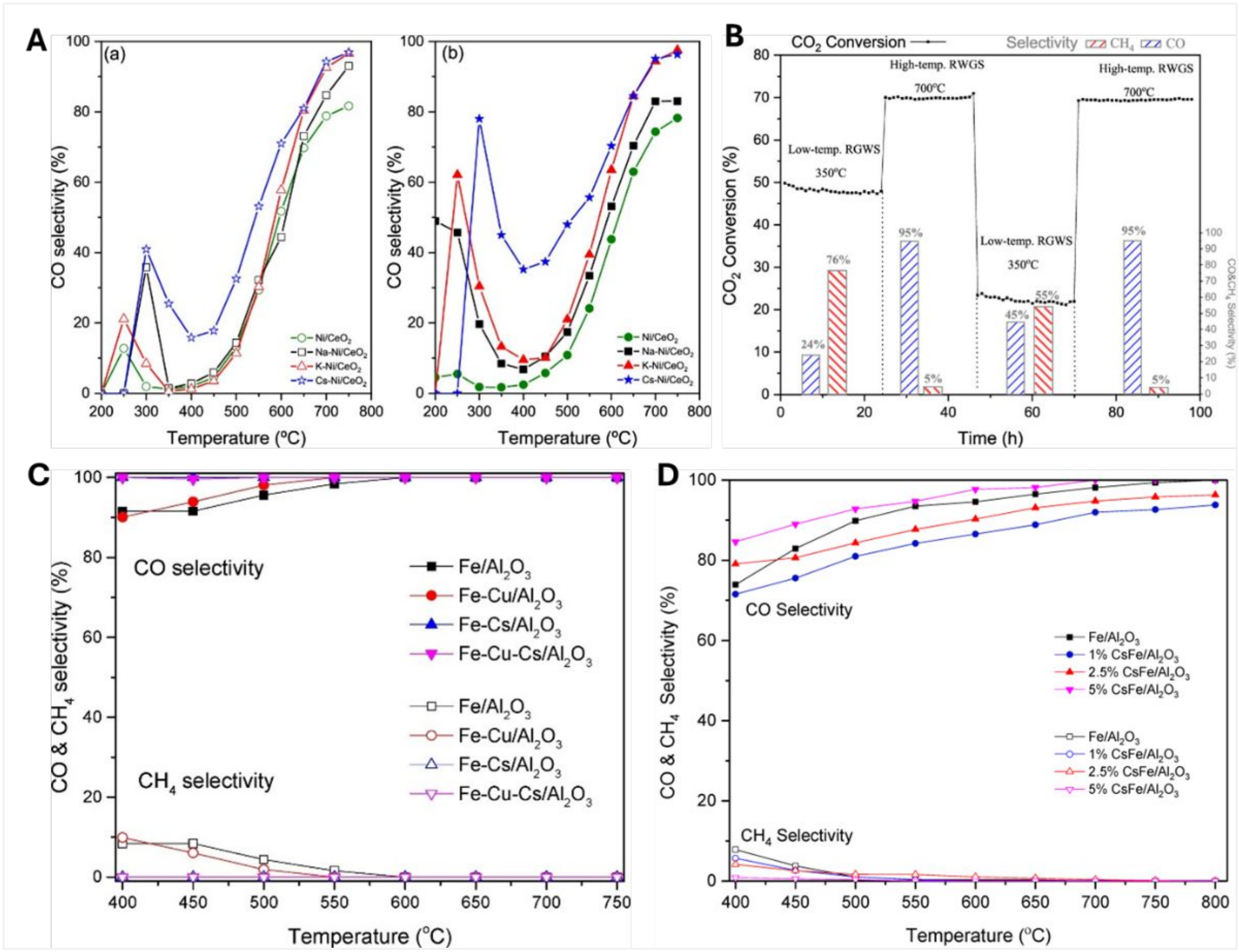

Figure S2: Catalytic activity and stability run for different Ni/CeO_2, $Fe\text{-}Cu/Al_2O_3$, and Fe/Al_2O_3-based catalysts. A-B) Reproduced with permission from ref. [2], Copyright 2022 American Chemical Society. C) Reproduced with permission from ref. [3], Copyright 2017 Elsevier. D) Reproduced with permission from ref. [4], Copyright 2018 MDPI.

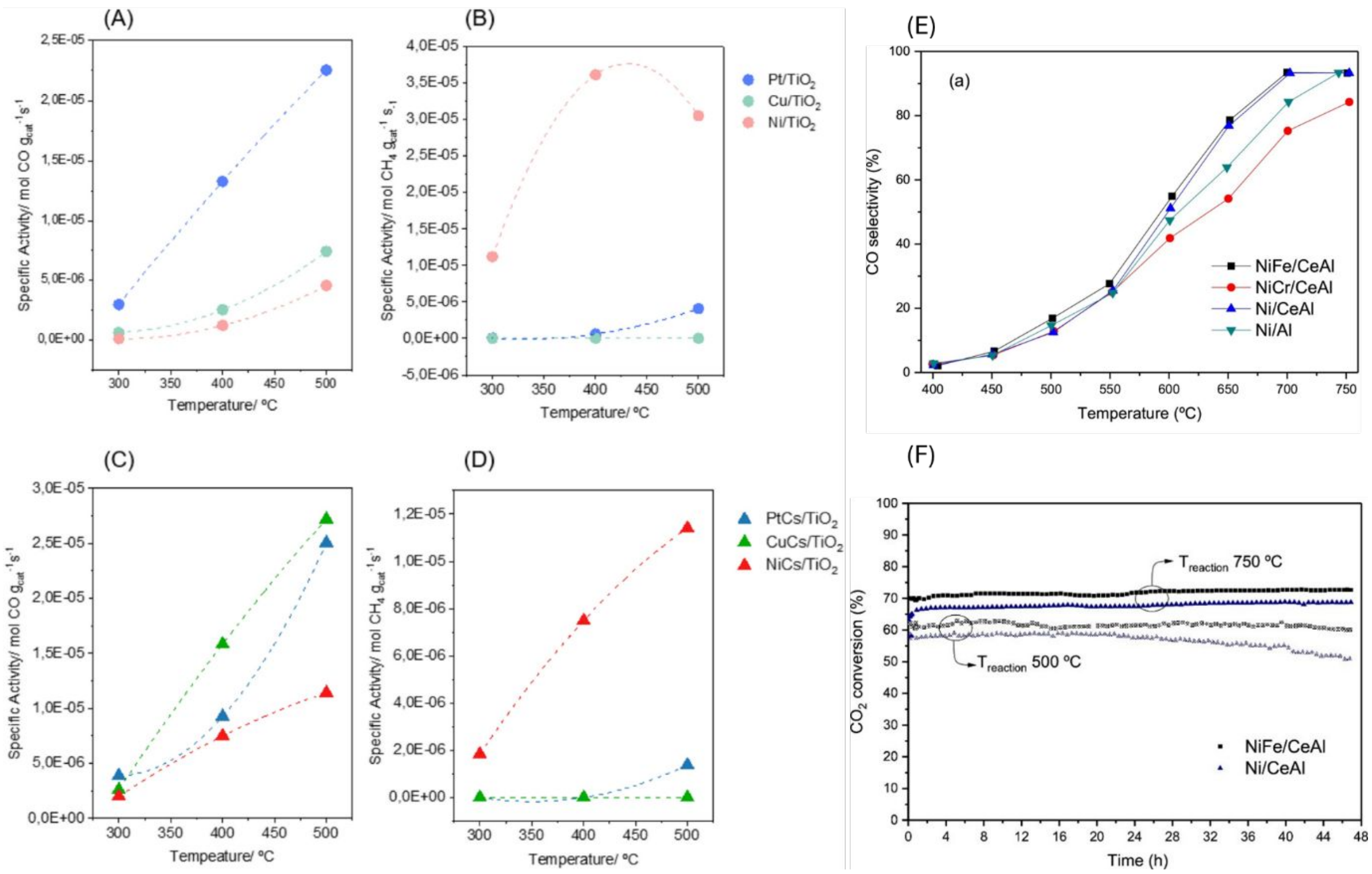

Figure S3: A-D) Specific activity towards CO and CH_4 for different Pt and Cs-doped catalysts, Reproduced with permission from ref. [5] Copyright 2024 SPRINGER NATURE. E-F) CO_2 conversion and stability tests of NiFe/CeAl and NiCr/CeAl catalysts at different conditions. Reproduced with permission from ref. [6] Copyright 2018 Elsevier.

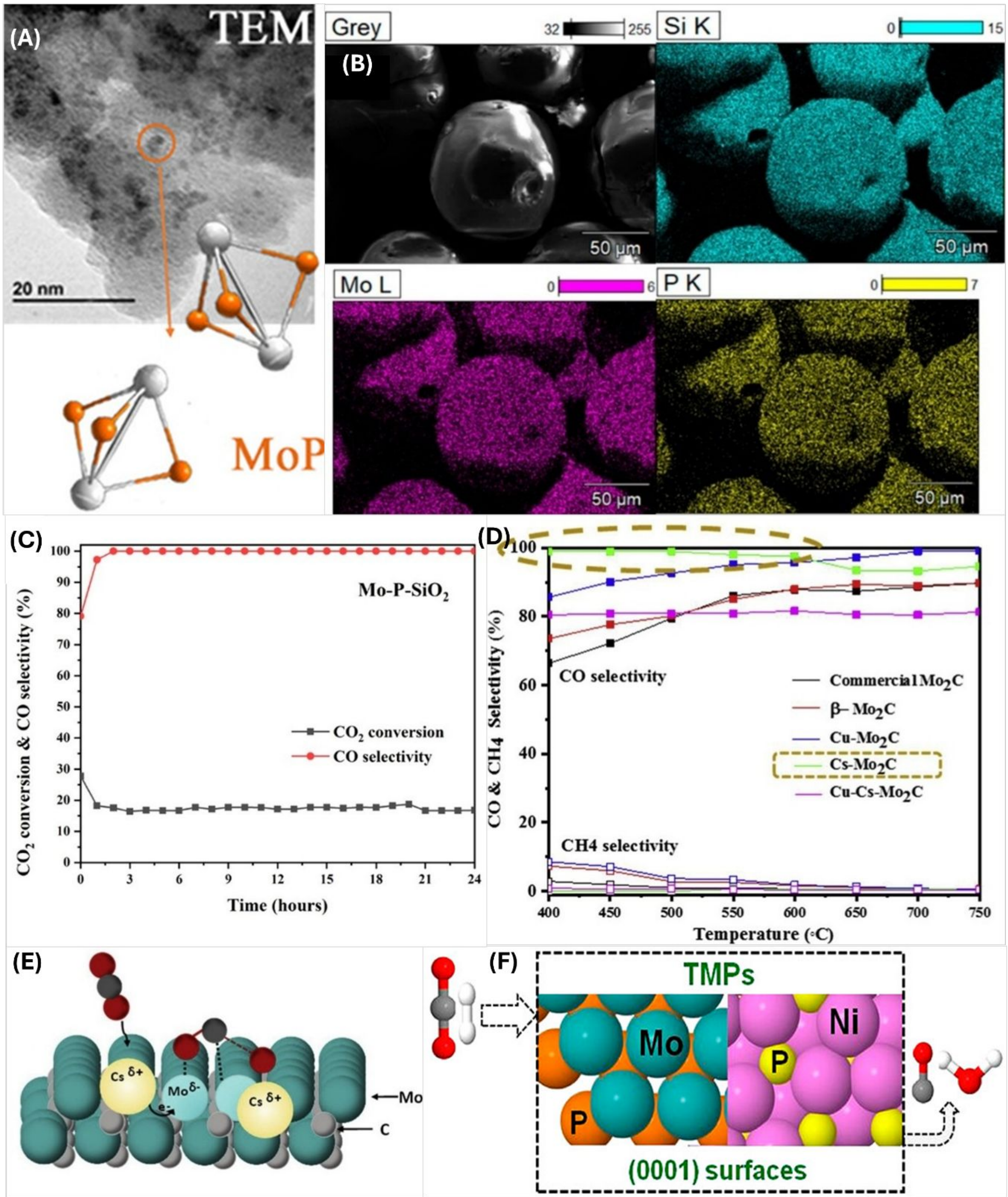

Figure S4: A-C) TEM micrographs, SEM micrographs with EDX analysis, and C) catalytic activity of Mo-P-SiO_2. Reproduced with permission from the ref. [7] Copyright 2022 ACS. D-E) Catalytic activity and schematic illustration of Cu and Cs promoted Mo_2C catalysts for RWGS, Reproduced with the permission from ref. [8] Copyright 2019 Elsevier. F) Comparative DFT illustration of MoP (0001) & Ni_2P (0001) surfaces for selected RWGS intermediates, Reproduced with the permission from ref. [9] Copyright 2019 ACS.

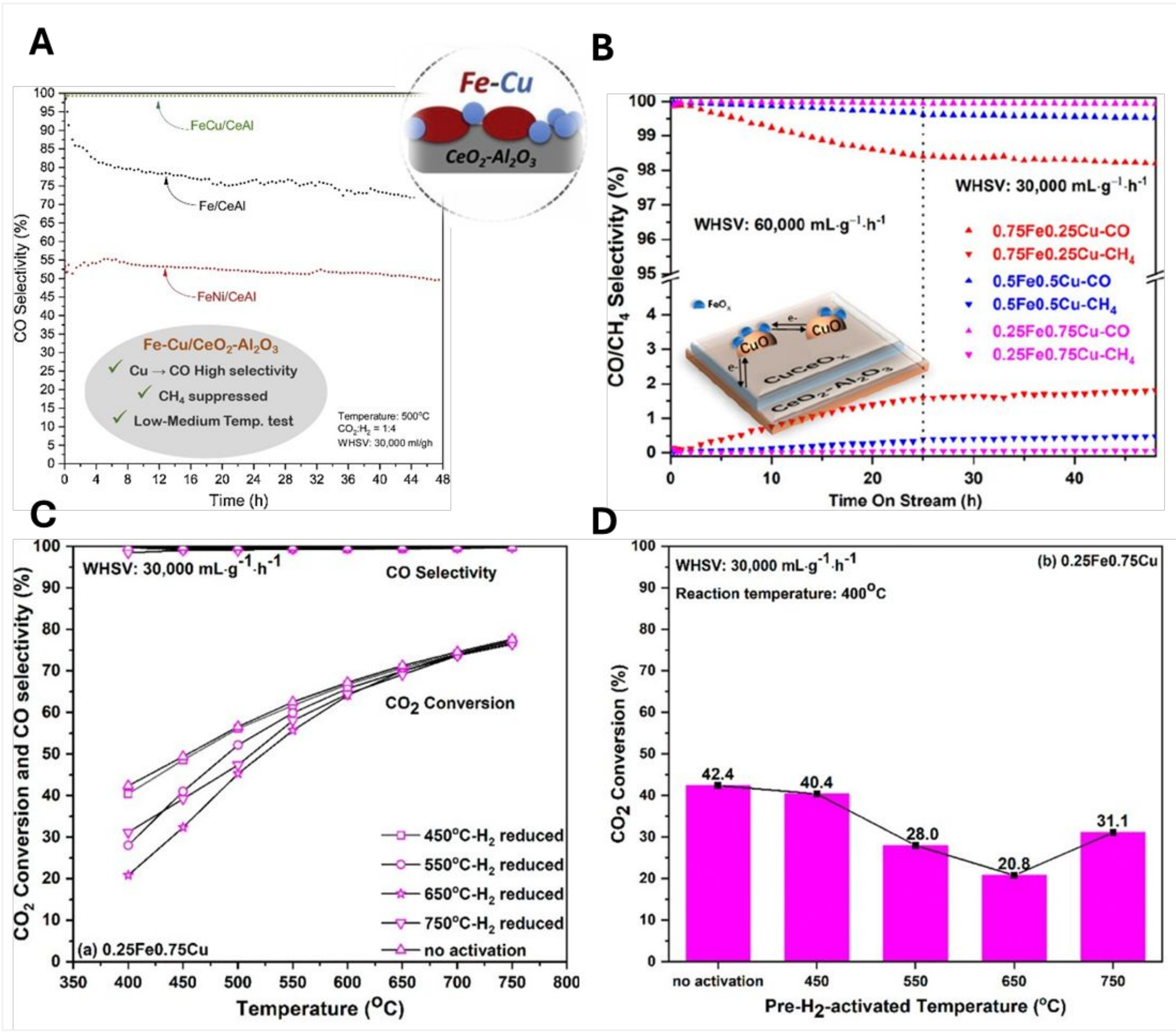

Figure S5: A) Stability test for Fe/CeAl, FeNi/CeAl and FeCu/CeAl Catalysts, Reproduced with permission from ref. [10], Copyright 2020 Elsevier. B) CO and CH_4 selectivity for Fe/Cu catalysts with different metal ratios, C-D) CO_2 conversion of 0.25Fe0.75Cu after different pre-H_2-activation temperatures. Reproduced with permission from ref. [11], Copyright 2021 American Chemical Society.

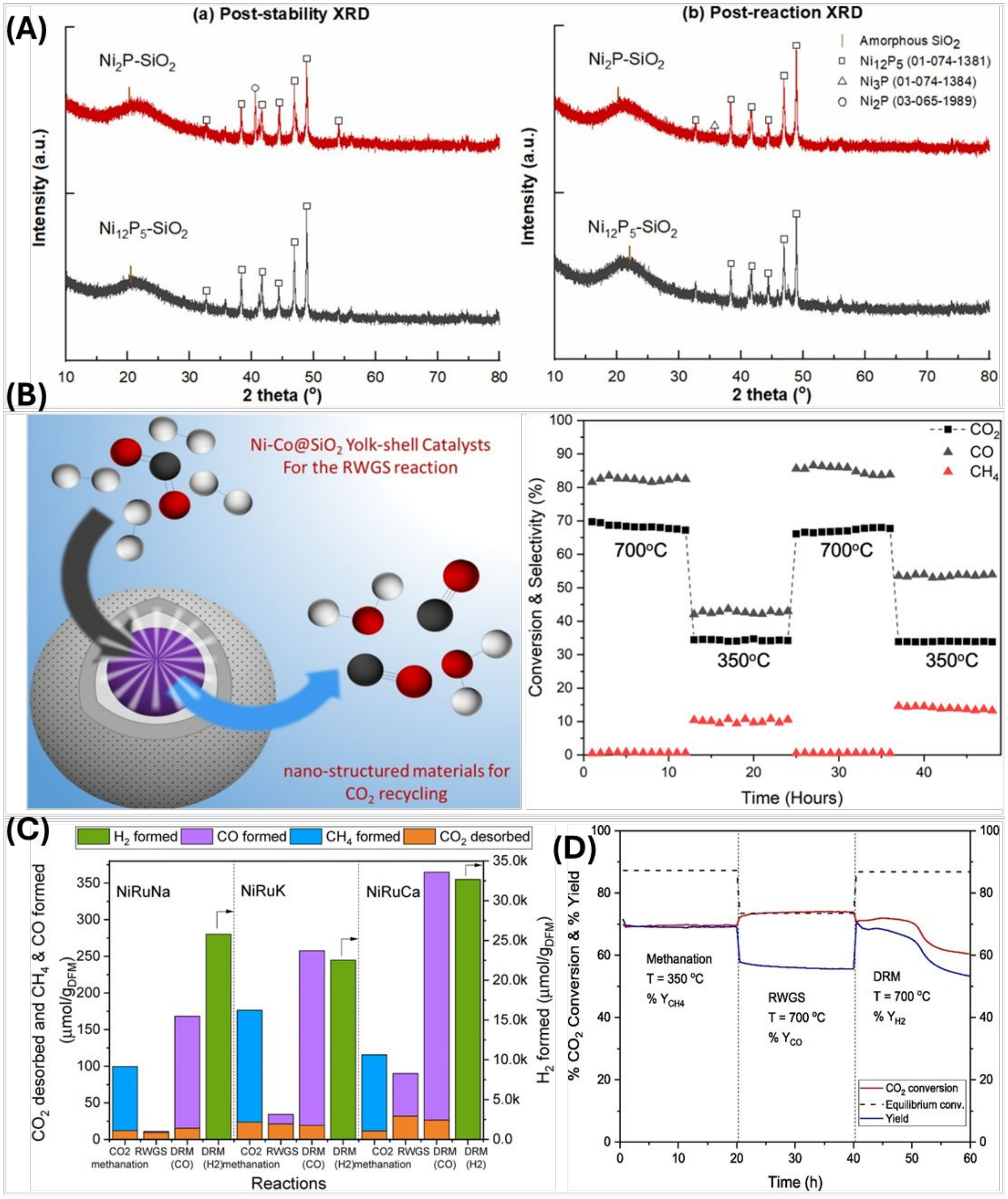

Figure S6: A) Post-stability and post-reaction XRD patterns for $Ni_{12}P_5$-SiO_2 and Ni_2P-SiO_2 catalysts. Reproduced with permission from ref. [12], Copyright 2023 Elsevier. B) Schematic illustration & cyclability study of the NiCo@SiO_2 catalyst at 15,000 ml/g_{cat}h, H_2:CO_2 = 4. Reproduced with permission from ref. [13], Copyright 2022 Elsevier. C) Feasibility study results of stable-switchable NiRuNa, NiRuK and NiRuCa DFMs. Reproduced with permission from ref. [14], Copyright 2022 Royal Society of Chemistry. D) Stability run for NiRu/CeAl in different

CO_2 conversion platforms (Methanation, RWGS, and DRM). Reproduced with permission from ref. [15], Copyright 2022 Elsevier.

References:

1. Rezaei, E.; Dzuryk, S. Techno-Economic Comparison of Reverse Water Gas Shift Reaction to Steam and Dry Methane Reforming Reactions for Syngas Production. *Chem. Eng. Res. Des.* **2019**, *144*, 354–369.
2. Gandara-Loe, J.; Zhang, Q.; Villora-Picó, J. J.; Sepúlveda-Escribano, A.; Pastor-Pérez, L.; Ramirez Reina, T. Design of Full-Temperature-Range RWGS Catalysts: Impact of Alkali Promoters on Ni/CeO_2. *Energy Fuels* **2022**, *36*, 6362–6373.
3. Pastor-Pérez, L.; Baibars, F.; le Saché, E.; Arellano-García, H.; Gu, S.; Reina, T. R. CO_2 Valorisation via Reverse Water-Gas Shift Reaction Using Advanced Cs-Doped Fe-Cu/Al_2O_3 Catalysts. *J. CO_2 Util.* **2017**, *21*, 423–428.
4. Pastor-Pérez, L.; Shah, M.; le Saché, E.; Ramirez Reina, T. Improving Fe/Al_2O_3 Catalysts for the Reverse Water-Gas Shift Reaction: On the Effect of Cs as Activity/Selectivity Promoter. *Catalysts* **2018**, *8*, 608.
5. Torres-Sempere, G.; González-Arias, J.; Penkova, A.; Santos-Muñoz, J. L.; Bobadilla, L. F.; Odriozola, J. A.; Pastor-Pérez, L.; Reina, T. R. CO_2 Conversion via Low-Temperature RWGS Enabled by Multicomponent Catalysts: Could Transition Metals Outperform Pt? *Top. Catal.* **2025**, *68*, 114–125.
6. Yang, L.; Pastor-Pérez, L.; Gu, S.; Sepúlveda-Escribano, A.; Reina, T. R. Highly Efficient Ni/CeO_2-Al_2O_3 Catalysts for CO_2 Upgrading via Reverse Water-Gas Shift: Effect of Selected Transition Metal Promoters. *Appl. Catal., B* **2018**, *232*, 464–471.
7. Zhang, Q.; Bown, M.; Pastor-Pérez, L.; Duyar, M. S.; Reina, T. R. CO_2 Conversion via Reverse Water Gas Shift Reaction Using Fully Selective Mo-P Multicomponent Catalysts. *Ind. Eng. Chem. Res.* **2022**, *61*, 12857–12865.
8. Zhang, Q.; Pastor-Pérez, L.; Jin, W.; Gu, S.; Reina, T. R. Understanding the Promoter Effect of Cu and Cs over Highly Effective β-Mo_2C Catalysts for the Reverse Water-Gas Shift Reaction. *Appl. Catal., B* **2019**, *244*, 889–898.
9. Guharoy, U.; Ramirez Reina, T.; Gu, S.; Cai, Q. Mechanistic Insights into Selective CO_2 Conversion via RWGS on Transition Metal Phosphides: A DFT Study. *J. Phys. Chem. C* **2019**, *123*, 22918–22931.
10. Yang, L.; Pastor-Pérez, L.; Villora-Pico, J. J.; Gu, S.; Sepúlveda-Escribano, A.; Reina, T. R. CO_2 Valorisation via Reverse Water-Gas Shift Reaction Using Promoted Fe/CeO_2-Al_2O_3 Catalysts: Showcasing the Potential of Advanced Catalysts to Explore New Process Design. *Appl. Catal., A* **2020**, *593*, 117442.
11. Yang, L.; Pastor-Pérez, L.; Villora-Pico, J. J.; Sepúlveda-Escribano, A.; Tian, F.; Zhu, M.; Han, Y. F.; Ramirez Reina, T. Highly Active and Selective Multicomponent Fe-Cu/CeO_2-Al_2O_3 Catalysts for CO_2 Upgrading via RWGS: Impact of Fe/Cu Ratio. *ACS Sustainable Chem. Eng.* **2021**, *9*, 12155–12166.
12. Hameed, G.; Goksu, A.; Merkouri, L. P.; Penkova, A.; Reina, T. R.; Ruiz, S. C.; Duyar, M. S. Experimental Optimization of Ni/P Atomic Ratio for Nickel Phosphide Catalysts in Reverse Water-Gas Shift. *J. CO_2 Util.* **2023**, *77*, 102606.

13. Price, C. A. H.; Pastor-Perez, L.; Reina, T. R.; Liu, J. Yolk-Shell Structured NiCo@SiO_2 Nanoreactor for CO_2 Upgrading via Reverse Water-Gas Shift Reaction. *Catal. Today* **2022**, *383*, 358–367.
14. Merkouri, L.-P.; Ramirez Reina, T.; Duyar, M. S. Feasibility of Switchable Dual Function Materials as a Flexible Technology for CO_2 Capture and Utilisation and Evidence of Passive Direct Air Capture. *Nanoscale* **2022**, *14*, 12620–12637.
15. Merkouri, L.-P.; le Saché, E.; Pastor-Pérez, L.; Duyar, M. S.; Ramirez Reina, T. Versatile Ni-Ru Catalysts for Gas Phase CO_2 Conversion: Bringing Closer Dry Reforming, Reverse Water Gas Shift and Methanation to Enable End-Products Flexibility. *Fuel* **2022**, *315*, 123097.
